# Development and analysis of a remimazolam pharmacokinetics and pharmacodynamics model with proposed dosing and concentrations for anaesthesia and sedation

**DOI:** 10.1016/j.bja.2025.02.038

**Published:** 2025-04-30

**Authors:** Douglas J. Eleveld, Pieter J. Colin, Johannes P. Van den Berg, Jeroen V. Koomen, Thomas Stoehr, Michel M.R.F. Struys

**Affiliations:** 1 Department of Anesthesiology, University Medical Center Groningen, University of Groningen, Groningen, The Netherlands; 2 Paion Pharma GmbH, Aachen, Germany; 3 Department of Basic and Applied Medical Sciences, Ghent University, Ghent, Belgium

**Keywords:** ADME, antagonism, CNS7054, drug interaction, metabolism, pharmacodynamics, pharmacokinetics, remimazolam

## Abstract

**Background:**

Pharmacokinetic–pharmacodynamic (PK–PD) models of remimazolam and their covariate relationships are useful for understanding drug disposition and predicting drug effects. Although clinical studies have shown that remimazolam induction doses decline with advancing age, this property is not reflected in existing models. The purpose of this investigation was to develop a PK–PD model for remimazolam and perform covariate analysis to maximise its utility across broad, diverse populations and to evaluate its consistency with clinical observations of drug dosing.

**Methods:**

Arterial and venous concentrations of remimazolam and its metabolite, Modified Observer's Assessment of Alertness and Sedation score and bispectral index were determined in 20 studies. Final population models were developed with covariate analysis. Simulations of drug administration for sedation and anaesthesia for this and previously published models were compared with the results of clinical studies.

**Results:**

Model development proceeded from 933 individuals aged 6–93 yr and weight 21–171 kg. PK data from studies with extracorporeal membrane oxygenation and treatment in ICU were considered in a *post hoc* analysis. Simulations of target-controlled infusion with the final model targeting sedation (Modified Observer's Assessment of Alertness and Sedation score 2 or 3) or anaesthesia (bispectral index 50) showed drug administration declining with age consistent with clinical observations.

**Conclusions:**

A PK–PD model for remimazolam was developed for a broad, diverse population. Dosing and target concentrations are proposed that are clinically useful for anaesthesia and sedation, especially for target-controlled infusion administration.

**Editor's key points**

• A pharmacokinetic–pharmacodynamic (PK–PD) model of remimazolam and its covariate relationships was developed to describe drug disposition and predict drug effects.
• Data from 20 studies (933 individuals) were used to model arterial and venous concentrations of remimazolam and its metabolite CNS7054, MOAA/S score, and BIS with covariate analysis and simulations of dosing for sedation and anaesthesia.
• The model identifies the influence of age, sex, concomitant opioid administration, hepatic and renal dysfunction, use of ECMO, and treatment in the ICU on the model, consistent with clinical studies.
• The model will facilitate TCI applications once clinically validated.

Remimazolam (Byfavo®, PAION Pharma GmbH, Aachen, Germany) is a water-soluble intravenous benzodiazepine with structural modifications similar to remifentanil. It is rapidly hydrolysed to a pharmacologically inactive metabolite (CNS7054) via carboxyl esterase 1^1^^,^^2^ which is present in most tissues with relatively higher levels in the liver.^3^ Remimazolam is indicated for procedural sedation (EU, USA, China, and South Korea) and general anaesthesia (EU, Japan, China, and South Korea) in adults.^4^, ^5^, ^6^

Pharmacokinetic (PK) models are useful for understanding drug disposition, and pharmacodynamic (PD) models are useful for predicting drug effects. Combined PK–PD models have potential application within target-controlled infusion (TCI) systems^7^ to optimise drug administration, although currently available systems are limited to three-compartment mammillary models. PK models can also be used for effect-site-targeted TCI if an associated effect-site rate constant, ke0, is known. If ke0 is not known, and cannot be calculated from the time of peak effect, plasma targeting must be used. The disadvantage of targeting the plasma concentration is that patients will respond more slowly to changes in target concentration than if the effect-site is targeted. For remimazolam, models meeting the criteria for effect-site targeting have appeared by Masui and colleagues,^8^ Masui and Hagihira,^9^ Schüttler and colleagues,^10^ Eisenried and colleagues,^11^ and Zhou and colleagues.^12^ Clinical studies have concluded that remimazolam induction doses should decline with advancing age^13^^,^^14^ but this property is not found in current PK or PD models. In addition to being scientifically unsatisfying, this discordance creates uncertainty in clinical applications because there is a lack of model-informed remimazolam concentrations suitable for clinical procedures across a broad, diverse clinical population.

We developed a PK–PD model for remimazolam useful across a broad, diverse population and evaluated its consistency with clinical observations of drug dosing and effects for sedation and anaesthesia. PK model development was driven by remimazolam and CNS7054 arterial and venous concentrations, and PD model development by drug effect measures of responsiveness to stimulation and electroencephalographic (EEG) measures of cerebral drug effect. Covariate analysis was performed to focus on those available in routine clinical applications such as age, weight, sex, etc. Model structures were considered that allow TCI applications. With the final model, we compared the expected remimazolam administration targeting sedation and anaesthesia with published dosing recommendations.

## Methods

Data were obtained from 20 studies, all of which were approved by the appropriate institutional ethics committees, and written informed consent obtained from all subjects before data collection, as declared by the original studies. All data were provided by PAION (owner of the data) or owned by UMCG (Groningen, The Netherlands). Studies included in the analysis are shown in Supplementary Table S1.

Before and during model development, some outlier observations were removed as likely erroneous. These included negative or zero concentrations, unexpected observations at unrealistic time points, and exceedingly high or unexpected very low concentration or drug effect observations. To be parsimonious, reduce sensitivity of the model to misspecification of front-end kinetics,^15^ and avoid issues with non-physiologically reasonable central compartment volumes,^16^ remimazolam and CNS7054 concentration observations were not included in the analysis for the first 2 and 3 min of drug administration, respectively.

Studies of TCI drug administration involve infusion profiles with frequent small changes in infusion rate, slowing data analysis. We combined sequential dose records if they were separated by <1 s, the infusion rates differed by <50 μg min^−1^, and infusion duration was <2 min. Consecutive records were merged with the summed dose over the combined duration until no dosing records could be combined. To verify that the simplified infusion profile was not distorted, it was checked that model fit was not significantly changed.

The Modified Observer's Assessment of Alertness and Sedation^17^ (MOAA/S) score is frequently used in quantifying drug effects on sedation, as shown in Supplementary Table S2, MOAA/S scores of 2–3 are targeted for procedural sedation, with lower levels required for induction of anaesthesia, for example MOAA/S scores of 0–1 for laryngoscopy and tracheal intubation.

Intraoperative anaesthetic drug effects can be quantified using the bispectral index (BIS) (Medtronic, Dublin, Ireland), a processed EEG measure. The data contained frequent BIS sampling, slowing data analysis. The number of BIS samples was reduced by median filtering in epochs of 10 s. Signal processing delay for the BIS signal was assumed to be 15 s. No distinction was made between different BIS monitor software versions used across studies.

### Model development

For PK model development, two- and three-compartment mammillary models were applied with parameters representing volumes (V1, V2, and V3), elimination clearance (CL), and intercompartmental CL (Q2 and Q3). Concentrations in the central compartment were associated with arterial observations while venous observations were associated with a first-order delayed (Kdelay_ref_) compartment as described by Hermann and colleagues^18^ with variations described by Henthorn and colleagues.^19^ The remimazolam fraction metabolised was assumed to be 100%^20^ appearing in a depot compartment and subsequently transferred to the CNS7054 central compartment by a first-order rate constant. The CNS7054 model was developed using the individualised estimates of the remimazolam PK model, known as the sequential method.^21^ This required individuals used for CNS7054 model development to have remimazolam samples.

Model parameters were (log) estimated relative to a reference individual, a 70-kg 35-yr-old male, in the absence of concomitant opioids, with normal hepatic and renal function. Allometric size scaling exponents of 1 for volumes and 0.75 for CL were used for the initial model, and different scaling methods were considered during model development.^22^ Peripheral components used compartmental allometry.^23^ Interindividual variability was assumed log-normally distributed, and PK residual observation error where a transform-both-sides approach was applied. Multiple sessions for an individual were treated as independent individuals. NONMEM version 7.5 (Icon Development Solutions, Ellicott City, MD, USA) was used for model estimation using the first-order conditional estimation method with interaction. For MOAA/S PD model estimation the LAPLACE option was used. Simulations and calculations of model predictive performance were performed using the R language^24^ version 3.4.4. Interindividual population variability is given as variance in the log-domain and coefficient of variation CV(%) calculated as: $\sqrt{\exp\left(\omega^{2}\;\right)-1}\cdot 100\%$. Estimation uncertainty was evaluated by likelihood profiles to determine upper and lower 99% confidence limits defined as the (spline interpolated) limits of each parameter required to increase NONMEM objective function by 6.63.

For covariate analysis, the *post hoc* eta *vs* covariate plots were examined, and statistically significant relationships (*P*<0.001, linear regression or Mann–Whitney test) with a reasonable mechanistic interpretation were subsequently tested for inclusion in the model. We considered the covariates: age, weight, sex, presence or absence of opioids, hepatic function (Pugh-Child score >8), and end-stage renal disease. Continuous covariates used an exponential model except for weight which used a power function. Binary covariates were implemented as a multiplicative factor. If indicators of hepatic and renal function were missing, normal hepatic and renal function were assumed. Adding a parameter to the model required a decrease in the Akaike Information Criteria (AIC) >13.8, which corresponds to a (two model comparison) relative likelihood^25^ of 0.001.

For MOAA/S and BIS PD model development, a similar sequential method was applied using individualised remimazolam and CNS7054 concentrations, which requires individuals used for PD model development to have remimazolam and CNS7054 samples. Both measures were independently linked to arterial remimazolam concentrations by an effect-site with a first-order time constant (ke0).

∂Ce/∂t=ke0·(Cp−Ce)

Tolerance to benzodiazepines is well known. To capture this behaviour a competitive interaction model^26^ was tested which has been suggested for remimazolam and CNS7054.^27^

MOAA/S observations were ordered categorical responses modelled using proportional odds with estimated cumulative probabilities of MOAA/S scores. For MOAA/S score S the logits l_x_ of the probabilities were: $l_{S=0}=b_{0}+\text{DEFF}\cdot \frac{{\left(\text{Ce}/\text{Ce}50\right)}^{\gamma }}{1+{\left(\text{Ce}/\text{Ce}50\right)}^{\gamma }+{\left(\text{Ca}/\text{Ca}50\right)}^{\gamma }},l_{S\leq 1}={l_{S=0}+b}_{1}$, $l_{S\leq 2}={l_{S\leq 1}+b}_{2}$, $l_{S\leq 3}={l_{S\leq 2}+b}_{3}$, $l_{S\leq 4}={l_{S\leq 3}+b}_{4}$, with corresponding probabilities ${\text{PC}}_{x}=\frac{e^{l_{x}}}{\left(1+e^{l_{x}}\right)}$ and the probability of observing a score are: $p_{S=0}={\text{PC}}_{S=0}$, $p_{S=1}={\text{PC}}_{S\leq 1}{-\text{PC}}_{S=0}$, $p_{S=2}={\text{PC}}_{S\leq 2}{-\text{PC}}_{S\leq 1}$, $p_{S=3}={\text{PC}}_{S\leq 3}{-\text{PC}}_{S\leq 2}$, $p_{S=4}={\text{PC}}_{S\leq 4}{-\text{PC}}_{S\leq 3}$, $p_{S=5}=1-{\text{PC}}_{S\leq 4}$. Here Cp is the remimazolam arterial concentration, Ce the effect-compartment concentration, Ca the CNS7054 arterial concentration, Ce50 the concentration for 50% remimazolam effect, and Ca50 the competitive interaction by CNS7054. Parameter b_0_ is a fixed effect parameter representing the logit of the probability for score 0 and b1 though b4 represent the difference in logits between the scores, and DEFF is a fixed effect parameter for the model.

BIS model development used a sigmoidal Emax equation for drug interaction and additive residual error (ε):

$$\text{BIS}=\text{BIS}\;\cdot \left(1-\frac{{\left(\text{Ce}/\text{Ce}50\right)}^{\gamma }}{1+{\left(\text{Ce}/\text{Ce}50\right)}^{\gamma }+{\left(\text{Ca}/\text{Ca}50\right)}^{\gamma }}\right)+\varepsilon$$

Evaluation of model predictive performance used the performance error (PE_PK_) and absolute performance error (APE_PK_)^28^ assuming proportional scaling for PK measures and additive scaling for BIS, reporting median values and prefixing with Md. For PK measures, we considered MdPE <10–20% and MdAPE between 20% and 40% to indicate clinically acceptable performance.^29^^,^^30^

### Simulations

The final PK–PD model was used to provide guidance for target concentrations likely suitable for the intended drug effects.^31^ For sedation, we simulated targeting a high probability of MOAA/S 2 or 3, and for anaesthesia a 90% probability of MOAA/S value of 0 at induction. The covariate relationships for the simulated individuals were from those used for model estimation or some example individuals. Simulations were for 60 min at a constant target concentration and assumed the presence of opioids and normal hepatic and renal function. The ke0 value for the TCI algorithm was that from the MOAA/S PD model and the target concentration was chosen as a factor of the estimated MOAA/S Ce50 value. For comparison with other models, we performed simulations of published remimazolam models that are applicable within TCI systems.

## Results

The aggregated data files contained data from 1930 individual sessions from 20 studies. Individuals without remimazolam concentrations were excluded from PK analysis. Exploratory analysis found that the studies CNS7056-010, ONO-2745-04, and REHSCU_ICU disrupted PK model development, likely because of the PK changes associated with extracorporeal membrane oxygenation (ECMO) and treatment in the ICU. We decided to develop a PK model without these studies and to consider them in a *post hoc* analysis. Demographic information of the 933 individuals analysed for PK model development is shown in Supplementary Figure S1.

### Remimazolam pharmacokinetic model development

For remimazolam PK model development, 7633 observations were available (3737 arterial, 3896 venous) from 933 individuals (568 male, 365 female). In the initial model. down-sampling of infusion records resulted in negligible changes in model fit and estimated parameters. Prediction of venous samples was judged to be suboptimal. The scaling parameter was fixed to 1 and a parameter added to predict venous concentrations as a fraction (Fven) between the delayed and central compartments, which resulted in improved model fit. Stepwise examination of *post hoc* eta plots and covariate testing resulted in improved model fit: V3 increased with increasing age (ΔAIC=−25.7), CL increased in females (ΔAIC=−43.3), V3 increased in females (ΔAIC=−19.6), CL decreased in the presence of concomitant opioids (ΔAIC=−27.5), and V3 increased with hepatic insufficiency (Pugh-Child score >8) (ΔAIC=−22.3). Variability of CL was found to be greater for venous only sampling (ΔAIC=−80.1).

This model showed good predictions with an MdAPE of 16.9% for arterial samples while venous samples were slightly worse at 26.0%. Diagnostic plots are shown in Supplementary Figure S2 (arterial samples) and Supplementary Figure S3 (venous samples). The likelihood profiles in Supplementary Figure S4 show the expected smooth approximately parabolic shape.

### CNS7054 pharmacokinetic model development

For CNS7054 PK model development, 7420 observations were available (3276 arterial, 4144 venous) from 905 individuals (541 male, 364 female). The arterial–venous model estimated for remimazolam was also applied to CNS7054. Stepwise examination of *post hoc* eta plots and covariate testing resulted in improved model fit: CL decreased with end-stage renal disease (ΔAIC=−134.4), an estimated allometric weight-scaling exponent (ΔAIC=−123.6), and in the presence of concomitant opioids (ΔAIC=−81.3). Q2 increased with hepatic insufficiency (Pugh-Child score >8) (ΔAIC=−15.8) and with increasing age (ΔAIC=−21.4).

This model showed good prediction of the observed CNS7054 concentrations with an MdAPE of 13.4% for arterial samples, and venous samples were slightly worse at 22.3%. Diagnostic plots are shown in Supplementary Figure S5 (arterial samples) and Supplementary Figure S6 (venous samples). Most parameters showed the expected smooth approximately parabolic shape of the likelihood profiles in Supplementary Figure S7, although some parameters show asymmetric uncertainty in regions of lower probability.

### MOAA/S pharmacodynamic model development

For MOAA/S PD model development, 12 670 observations were available from 449 individuals (292 male, 157 female). The proportional odds MOAA/S model was extended to shift probability density from MOAA/S score 1 toward 0 in the presence of opioids which improved model fit (ΔAIC=−875.6). A sigmoidal drug effect showed considerable improvement in model fit (ΔAIC=−4058.9). Adding interindividual variability to Ce50 improved the model (ΔAIC=−4674.0) and a competitive antagonist model for the role of CNS7054 (ΔAIC=−394.6). Stepwise examination of *post hoc* eta plots and covariate testing resulted in improved model fit: sigmoidal drug effect Ce50 decreasing with age (ΔAIC=−31.2), and removal of the theoretical scaling exponent for ke0 (ΔAIC=−26.4). Use of an effect-site for CNS7054 did not improve model fit. The predicted relationship between effect-site concentration and MOAA/S score is shown in Supplementary Figure S8. The likelihood profiles in Supplementary Figure S9 show the expected smooth approximately parabolic shape.

### Bispectral index pharmacodynamic model development

For BIS PD model development, 118 833 observations were available from 613 individuals (355 male, 258 female). The initial BIS model had interindividual variability on Ce50 and ke0. Adding baseline variability equal to the residual variability improved the model (ΔAIC=−12 429.0), and a competitive antagonist model for the role of CNS7054 (ΔAIC=−20 126.7). Stepwise examination of *post hoc* eta plots and covariate testing resulted in improved model fit: sigmoidal drug effect Ce50 decreasing with age (ΔAIC=−165.5), and ke0 decreasing with age (ΔAIC=−34.8) and removal of the theoretical scaling exponent for ke0 (ΔAIC=−15.99). Use of an effect-site for CNS7054 did not improve model fit. The model shows some bias for population predictions <BIS 50. Other model modifications were tried but were judged not to improve model fit: lower limit to BIS (lower bound 0), increased delay (lower bound 15 s), and estimation of γ of PD sigmoid. We accepted this as the final model which had an MdAPE of 6.9 (BIS). Diagnostic plots are shown in Supplementary Figure S10. The likelihood profiles in Supplementary Figure S11 showed the expected smooth approximately parabolic shape.

### Final pharmacokinetic–pharmacodynamic model

**Fig 1:**
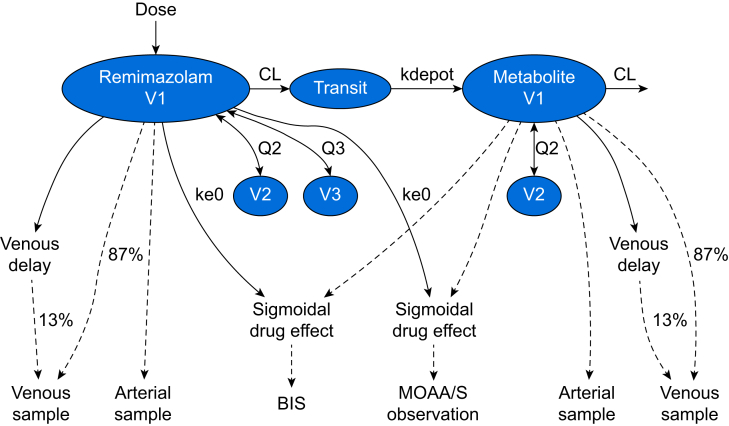
Final pharmacokinetic and pharmacodynamic model structure. BIS, bispectral index; CL, clearance; ke0, first-order time constant; MOAA/S, Modified Observer's Assessment of Alertness and Sedation.

A graphical representation of the final PK–PD model is shown in Figure 1, and the final parameter estimates are shown in Table 1. The model equations in NONMEM code are provided in the supplementary content. For remimazolam the model equations are:

$$F_{\text{size}}=\frac{\text{WGT}}{70}$$

$$F_{x∼\text{age}}=\exp\left(\frac{K_{x∼\text{age}}}{1000}\cdot \left(\text{AGE}-35\right)\right)$$

$$F_{x∼\text{sex}}=\exp\left(\frac{K_{x∼\text{sex}}}{100}\right)\;\text{if}\;\text{female},\text{otherwise}\;1$$

$$F_{x∼\text{opiates}}=\exp\left(\frac{K_{x∼\text{opiates}}}{100}\right)\;\text{if}\;\text{opiates}\;\text{present},\text{otherwise}\;1$$

$$F_{x∼\text{Pugh}}=\exp\left(\frac{K_{x∼\text{Pugh}}}{100}\right)\;\text{for}\;\text{Pugh}-\text{Child}\;\text{score}\;>\;8,\text{otherwise}\;1$$

$$F_{\omega^{2}\text{CL}∼\text{ven}}=K_{\omega^{2}\text{CL}∼\text{ven}}\;\text{for}\;\text{only}\;\text{venous}\;\text{samples},\text{otherwise}\;1$$

$$V1={V1}_{\text{ref}}\cdot F_{\text{size}}\cdot \exp\left(\omega_{V1}^{2}\right)$$

$$V2={V2}_{\text{ref}}\cdot F_{\text{size}}\cdot \exp\left(\omega_{V2}^{2}\right)$$

$$V3={V3}_{\text{ref}}\cdot F_{\text{size}}\cdot F_{V3∼\text{age}}\cdot F_{V3∼\text{sex}}\cdot F_{V3∼\text{Pugh}}\cdot \exp\left(\omega_{V3}^{2}\right)$$

$$\text{CL}={\text{CL}}_{\text{ref}}\cdot {F_{\text{size}}}^{0.75}\cdot F_{\text{CL}∼\text{sex}}\cdot F_{\text{CL}∼\text{opiates}}\cdot \exp\left(\omega_{\text{CL}}^{2}\cdot F_{\omega^{2}\text{CL},\text{ven}}\right)$$

$$Q2={Q2}_{\text{ref}}\cdot {\left(\frac{V2}{{V2}_{\text{ref}}}\right)}^{0.75}\cdot \exp\left(\omega_{Q2}^{2}\right)$$

$$Q3={Q3}_{\text{ref}}\cdot {\left(\frac{V3}{{V3}_{\text{ref}}}\right)}^{0.75}\cdot \exp\left(\omega_{Q3}^{2}\right)$$

**Table 1:** Final model parameter estimates. BIS, bispectral index; Concentration for 50% competitive interaction (Ca50); Concentration for 50% effect (Ce50); CL, clearance; CV, coefficient of variation; DEFF, drug effect multiplier; Fven, fraction; Kdelay_ref_, first-order delayed; ke0, first-order time constant; MOAA/S, Modified Observer's Assessment of Alertness and Sedation; ∗Not estimated directly but calculated from b0, d01 and K_do1_ when opioids were present.

Remimazolam	Estimated	Lower 1%	Upper 99%	IIV (*ω*^2^)	CV(%)
V1_ref_ (L)	4.31	3.98	4.66	0.0631	25.5
V2_ref_ (L)	12.3	11.2	13.5	0.203	47.5
V3_ref_ (L)	18.6	17.2	20.2	0.138	38.4
CL_ref_ (L min^−1^)	1.12	1.08	1.16	0.0297	17.4
Q2_ref_ (L min^−1^)	1.45	1.33	1.59	0.105	33.2
Q3_ref_ (L min^−1^)	0.298	0.267	0.332	0.118	35.5
Fven (%)	13.2	9.60	17.2		
Kdelay_ref_ (min^−1^)	0.0144	0.0110	0.0180		
K_V3∼age_	7.31	3.59	11.07		
K_CL∼sex_	16.3	11.0	21.6		
K_V3∼sex_	28.7	14.5	42.6		
K_CL∼opiates_	−13.9	−19.0	−8.87		
K_V3∼Pugh_	82.4	40.0	125		
K_*ω*_^2^_CL∼ven_	1.86	1.58	2.19		

**CNS7054**	**Estimated**	**Lower 1%**	**Upper 99%**	**IIV (*ω*^2^)**	**CV(%)**

K_depot_ (min^−1^)	0.215	0.198	0.234	0.257	54.2
V1_ref_ (L)	6.85	6.46	7.16	0.0888	30.5
V2_ref_ (L)	5.12	4.59	5.68	0.443	74.7
CL_ref_ (L min^−1^)	0.0665	0.0613	0.0721	0.145	39.5
Q2_ref_ (L min^−1^)	0.141	0.122	0.174	0.989	130
K_CL∼renal_	−218	-261	−179		
Kscale	0.518	0.424	0.613		
K_CL∼opiates_	−32.8	−43.1	−22.5		
K_Q2∼Pugh_	188	61.1	343		
K_Q2∼age_	15.7	10.5	24.6		

**MOAA/S**	**Estimated**	**Lower 1%**	**Upper 99%**	**IIV (*ω*^2^)**	**CV(%)**

DEFF	19.9	19.0	20.8		
ke0 (min^−1^)	0.298	0.290	0.310		
b0	−16.6	−17.7	−15.5		
d01	2.65	2.40	2.92		
b0 (opioids)	−14.4∗				
d01 (opioids)	0.500∗				
d12	0.942	0.867	1.02		
d23	1.47	1.38	1.55		
d34	2.54	2.41	2.66		
Ce50_ref_ (μg ml^−1^)	0.182	0.151	0.221	0.195	46.3
Ca50 (μg ml^−1^)	11.9	10.0	14.3		
K_Ce50∼age_	−7.63	−11.0	−4.31		
K_d01∼opiates_	1.67	1.52	1.82		

**BIS**	**Estimated**	**Lower 1%**	**Upper 99%**	**IIV (*ω*^2^)**	**CV(%)**

ke0_ref_ (min^−1^)	0.145	0.128	0.164	0.549	85.5
Ce50_ref_ (μg ml^−1^)	0.982	0.909	1.06	0.228	50.6
Baseline	93.7	92.9	94.5		
Ca50 (μg ml^−1^)	8.41	8.15	8.68		
K_Ce50∼age_	−16.4	−19.4	−13.4		
K_ke0∼age_	−10.6	−15.5	−5.86		

For CNS7054 the model equations are:

$$F_{\text{size}}={\left(\frac{\text{WGT}}{70}\right)}^{\text{Kscale}}$$

$$F_{x,\text{renal}}=\exp\left(\frac{K_{x,\text{renal}}}{100}\right)\;\text{end}-\text{stage}\;\text{renal}\;\text{disease},\text{otherwise}\;1$$

$$V1={V1}_{\text{ref}}\cdot F_{\text{size}}\cdot \exp\left(\omega_{V1}^{2}\right)$$

$$V2={V2}_{\text{ref}}\cdot F_{\text{size}}\cdot \exp\left(\omega_{V2}^{2}\right)$$

$$\text{CL}={\text{CL}}_{\text{ref}}\cdot {F_{\text{size}}}^{0.75}\cdot F_{\text{CL}∼\text{renal}}\cdot F_{\text{CL}∼\text{opiates}}\cdot \exp\left(\omega_{\text{CL}}^{2}\right)$$

$$Q2={Q2}_{\text{ref}}\cdot {\left(\frac{V2}{{V2}_{\text{ref}}}\right)}^{0.75}\cdot F_{\text{CL}∼\text{age}}\cdot F_{\text{CL}∼\text{Pugh}}\cdot \exp\left(\omega_{Q2}^{2}\right)$$

For MOAA/S the model equations are:

$$\text{ke}0={\text{ke}0}_{\text{ref}}$$

$$\text{Ce}50={\text{Ce}50}_{\text{ref}}\cdot F_{\text{Ce}50∼\text{age}}\cdot \exp\left(\omega_{\text{Ce}50}^{2}\right)$$

For BIS the model equations are:

$$\text{ke}0={\text{ke}0}_{\text{ref}}\cdot F_{\text{ke}0∼\text{age}}\cdot \exp\left(\omega_{\text{ke}0}^{2}\right)$$

$$\text{Ce}50={\text{Ce}50}_{\text{ref}}\cdot F_{\text{Ce}50∼\text{age}}\cdot \exp\left(\omega_{\text{Ce}50}^{2}\right)$$

where WGT is total body weight in kg, AGE is age in yr. The factors 100 and 1000 in the covariate functions are arbitrary and serve to ease NONMEM numerical issues by ensuring similar scales to the estimated parameters. To ease practical applications such as TCI, simplified equations for the remimazolam population predictions assuming normal hepatic function are provided:

KOCL=−0.139340∗(OA1P2-1)

KSV3=0.287037∗(M1F2-1)

KSCL=0.162802∗(M1F2-1)

KAV3=0.00730817∗(AGE-35)

VSIZ=WGT/70

CSIZ=(WGT/70.)∗∗0.75

V1=VSIZ∗4.30730

V2=VSIZ∗12.2994

V3=VSIZ∗18.6411∗exp(KAV3+KSV3)

CL=CSIZ∗1.11977∗exp(KSCL+KOCL)

Q2=(VSIZ∗∗0.75)∗1.45260

Q3=((V3/18.6411)∗∗0.75)∗0.297838

Ke0=0.298269

where M1F2 is male=1, female=2 and OA1P2 is opioid absent=1, present=2.

#### *Post hoc* analysis

For patients receiving ECMO, the final model performed poorly. The model was improved by including changes suggested by Zhou and colleagues,^12^ an increased V1 and decreased CL between the start and end of ECMO (ΔAIC=−1580.85). Treated as a group, these individuals showed equal decreases in remimazolam V1 and V2 (ΔAIC=−507.97), decreased remimazolam CL (ΔAIC=−50.35), increased CNS7054 peripheral volume (ΔAIC=−25.03), decreased remimazolam V3 (ΔAIC=−20.70), and CNS7054 CL decreased the same as for remimazolam (ΔAIC=−11.91). Removal of decreased CL between the start and end of ECMO resulted a negligible change in model fit (ΔAIC=1.93) and no other changes were judged to improve model fit. The MdAPE for arterial samples for remimazolam was 19.0% and for CNS7054 15.1%. Diagnostic plots for remimazolam are shown in Supplementary Figure S12 (arterial samples) and for CNS7054 in Supplementary Figure S13 (arterial samples). The likelihood profiles in Supplementary Figure S14 show the expected smooth approximately parabolic shape although some parameters show asymmetric uncertainty in regions of lower probability.

For individuals receiving treatment in the ICU, the final model performed poorly. The model was improved with increased remimazolam V3 (ΔAIC=−218.71), lower CNS7054 CL (ΔAIC=−328.20), and further lower remimazolam CL after 24 h (ΔAIC=−1494.85). No other changes were judged to improve model fit but predictive performance was poor, especially for venous samples. For remimazolam, MdAPE for arterial samples was 24.4% and venous samples were considerably worse at 83.7%. For CNS7054, MdAPE for arterial samples was poor at 45.8% and worse for venous at 66.6%. Diagnostic plots for remimazolam are shown in Supplementary Figure S15 (arterial samples) and Supplementary Figure S16 (venous samples), and for CNS7054 in Supplementary Figure S17 (arterial samples) and Supplementary Figure S18 (venous samples). The likelihood profiles in Supplementary Figure S19s show the expected smooth approximately parabolic shape.

The model covariate adjustment functions for individuals receiving ECMO and individuals receiving treatment in the ICU are:

$$F_{x∼\text{ECMO}}=K_{x∼\text{ECMO}}\;\text{during}\;\text{ECMO},\text{otherwise}\;1$$

$$F_{x∼I\text{ECMO}}=K_{x∼\text{IECMO}}\;\text{for}\;\text{individuals}\;\text{receiving}\;\text{ECMO},\text{otherwise}\;1$$

$$F_{x∼\text{ICU}}=K_{x∼\text{ICU}}\;\text{for}\;\text{individuals}\;\text{receiving}\;\text{treatment}\;\text{in}\;\text{ICU},\text{otherwise}\;1$$

$$F_{x∼24h}=K_{x∼24h}\;\text{for}\;\text{treatment}\;\text{in}\;\text{ICU}\;\text{longer}\;\text{than}\;24\;\text{hours},\text{otherwise}\;1$$

For remimazolam the model equations requiring adjustment are:

$$V1={V1}_{\text{PK}}\cdot F_{V1∼\text{ECMO}}\cdot F_{V∼\text{IECMO}}$$

$$V2={V2}_{\text{PK}}\cdot F_{V∼\text{IECMO}}$$

$$V3={V3}_{\text{PK}}\cdot F_{V3∼\text{IECMO}}\cdot F_{V3∼\text{ICU}}$$

$$\text{CL}={\text{CL}}_{\text{PK}}\cdot F_{\text{CL}∼\text{IECMO}}\cdot F_{\text{CL}∼24h}$$

For CNS7054 the model equations requiring adjustment are:

$$V2={V2}_{\text{PK}}\cdot F_{V2∼\text{IECMO}}$$

$$\text{CL}={\text{CL}}_{\text{PK}}\cdot F_{\text{CL}∼\text{IECMO}}\cdot F_{\text{CL}∼\text{ICU}}$$

where subscript PK indicates the calculated parameter from the previously developed PK model. The estimated model parameters are shown in Table 2.

**Table 2:** Adjustments to the remimazolam and CNS7054 pharmacokinetic model estimates for individuals receiving ECMO or treated in the ICU. CL, clearance; ECMO, extracorporeal membrane oxygenation.

	Estimated	Lower 1%	Upper 99%
K_V1∼ECMO_	46.1	35.2	63.2
K_V∼IECMO_	0.189	0.136	0.248
K_V2∼IECMO_	2.03	1.57	2.61
K_V3∼IECMO_	0.722	0.618	0.867
K_V3∼ICU_	1.59	1.32	1.97
K_CL∼IECMO_	0.815	0.768	0.864
K_CL∼24h_	0.264	0.239	0.295
K_CL∼ICU_	0.426	0.370	0.480

### Simulations

**Fig 2:**
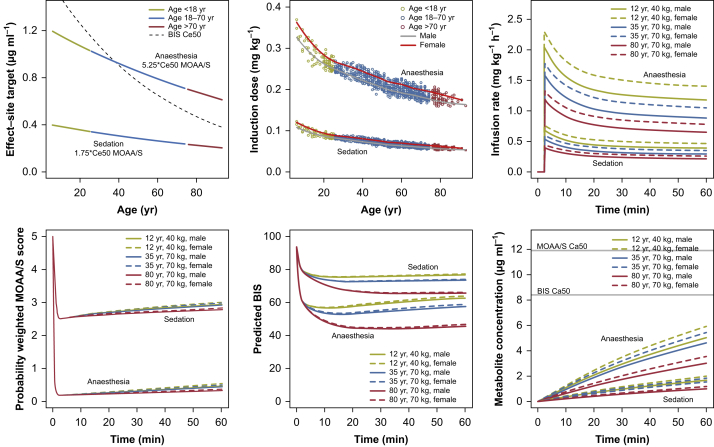
Simulations of effect-site TCI with the final model for sedation (MOAA/S 2 or 3) and anaesthesia (>90% MOAA/S 0), and predicted drug administration, concentrations and MOAA/S and BIS effects. BIS, bispectral index; Concentration for 50% drug effect (Ce50); MOAA/S, Modified Observer's Assessment of Alertness and Sedation; TCI, target-controlled infusion.

The results of simulations of TCI targeting sedation and anaesthesia are shown in Figure 2. The intended MOAA/S values were achieved after induction but drift higher over time despite a constant remimazolam concentration, an apparent development of tolerance. The degree of drift is greater for younger compared with older individuals. The effect-site target concentrations for these effects decrease with age for both sedation and anaesthesia and the induction doses and maintenance infusion rates decrease similarly. The influence of age in the BIS PD models is stronger than in the MOAA/S model, which can be seen comparing the BIS Ce50 to the effect-site targets for the simulations. It is also evident that the predicted BIS values are higher for younger individuals even as the predicted probability weighted MOAA/S scores are nearly equal around the time of induction. The difference here could point to differing mechanisms by which benzodiazepines influence EEG measures *vs* tolerance to stimulation.

**Fig 3:**
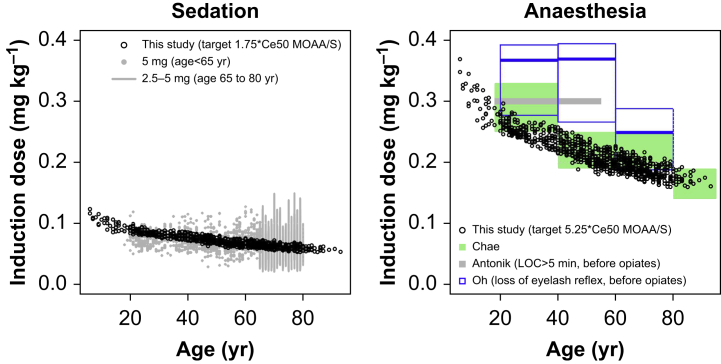
Simulations of effect-site TCI for sedation and anaesthesia comparing drug administration with recommendations from the literature. Concentration for 50% drug effect (Ce50); LOC, loss of consciousness; MOAA/S, Modified Observer's Assessment of Alertness and Sedation; TCI, target-controlled infusion.

A comparison of dosing from simulations to recommendations from the literature is shown in Figure 3. For sedation, the US Food and Drug Agency (FDA) drug label,^32^ European Medicine Agency (EMA) summary of product characteristics (SmPC), and other studies^33^^,^^34^ suggest doses of 5 mg in the presence of opioids, which corresponds to ∼0.07 mg kg^−1^ for a 70 kg individual. The EMA SmPC suggests a reduced dose of 2.5–5 mg for individuals 65–80 yr old. For anaesthesia, Chae and colleagues^13^ suggested doses of 0.25–0.33, 0.19–0.25, 0.14–0.19 mg kg^−1^ are suitable for induction of anaesthesia in patients aged <40, 60–80, >80 yr, respectively. Oh and colleagues^14^ found the 95% effective dose of remimazolam for induction of general anaesthesia (before opioid administration) was 0.367 mg kg^−1^, 95% confidence interval (CI) 0.277–0.392 mg kg^−1^ for age 20–40 yr, 0.369 mg kg^−1^, 95% CI 0.266–0.394 mg kg^−1^for 40–60 yr, and 0.249 mg kg^−1^, 95% CI 0.199–0.288 mg kg^−1^ for 60–80 yr. Antonik and colleagues^35^ found that 0.3 mg kg^−1^ causes loss of consciousness for >5 min in >50% of subjects 18–55 yr. This is higher than our simulations but not unexpected given a dose-dependent duration of effect, and our model showing a time to reach peak effect of about 2.5 min (Supplementary Fig. S20), whereupon maintenance infusion starts, so the total dose over 5 min will be larger. Our simulations assume the presence of opioids, but this was not always the case for other studies. Our model predicts lower CL in the presence of opioids, and this might moderately affect induction doses. One might expect a degree of concurrence provided that responsiveness is determined by nonpainful stimuli (MOAA/S values ≥2).

**Fig 4:**
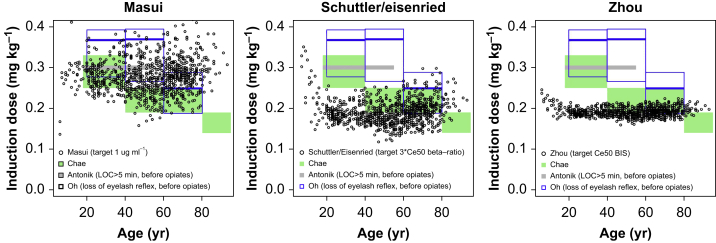
Simulations of effect-site TCI for anaesthesia comparing drug administration for the Masui, Schuttler and Eisenried, and Zhou (without remifentanil or cumulative dose interaction or time-dependant clearance) models. BIS bispectral index; Concentration for 50% drug effect (Ce50); LOC, loss of consciousness.

Simulations of TCI dosing were also performed for the models described by Masui^8^, Schüttler and Eisenried^11^, and Zhou^12^ (omitting remifentanil or cumulative dose interaction or time-dependant CL) for anaesthesia with targets obtained from the papers (Fig. 4). The calculated induction doses for these models do not match the age-dependant dosing found in clinical studies.

## Discussion

We developed a PK–PD model to predict remimazolam and CNS7054 arterial and venous concentrations and MOAA/S and BIS for a broad population. The model identifies the impact of age, sex, concomitant opioid administration, hepatic and renal dysfunction, use of ECMO, and treatment in the ICU on the model. The model structure allows for TCI applications in infusion pumps. In contrast to previous models, the model is consistent with clinical studies reporting that equal drug effects in sedation or anaesthesia require reduced remimazolam induction dose and maintenance infusion rate with increasing age.

The final PK model has broad similarities with reported models with central compartment volumes of 3–4 L, elimination rates of ∼1.1 L min^−1^, and peripheral compartment volumes of 10–30 L. The predicted time to peak effect is about 2.5 min, which is consistent with the results of Rex and colleagues^33^ who found 3.0 min (95% CI 2.0–3.0 min), Antonik and colleagues^35^ (1–4 min), and Masui and Hagihira^9^ (2.6 min). We found remimazolam elimination CL is increased in females, and Masui and colleagues^8^ and Zhou and colleagues^12^ found similar sex differences. This has also been observed for propofol^36^ and remifentanil^37^ and might be related to sex differences in lean liver volume.^38^

The presence of opioids is associated with ∼13% lower clearance (CL) of remimazolam. The mechanism is not clear, but could be related to dependence of elimination CL on cardiac output and liver blood flow, which might be reduced in the presence of opioids. Thus for a given target concentration, the presence of opioids moderately reduces the dose necessary to achieve a given target concentration.

In our MOAA/S model we took into account that opioids are analgesics and increase tolerance of noxious stimuli by shifting the probability of MOAA/S scores from 1 to 0 with other scores unchanged. Higher MOAA/S scores (5–3) are based on response to a verbal command and represent more the hypnotic component of anaesthesia for which opioid analgesics are less impactful. With the current model, sedation targeting MOAA/S scores 2 and 3 does not require different target concentrations in the absence of opioids, although dosing will be slightly higher because of predicted higher drug CL. When deep levels of sedation to MOAA/S scores 0 and 1 are required in the absence of opioids, higher target concentrations are likely necessary, further increasing the required dose compared with the presence of opioids.

Our data included observations from children as young as 6 yr old, and there were considerably fewer individuals under the age of 20 yr compared with adults. The PK model incorporates allometric scaling, which extrapolates from adults to children with lower doses but slightly higher doses calculated on a per-bodyweight basis. This is in line with clinical experience for many anaesthetics,^22^ supporting reasonable extrapolation properties of the model.

Patients treated with ECMO show strong changes to drug distribution volumes and decreased CL, likely attributable to patient clinical factors and properties of the ECMO procedure. For patients being treated in the ICU, our analysis indicates increased distribution volume, low CNS7054 CL, and a decrease in remimazolam CL for administration >24 h similar to the results of Zhou and colleagues.^12^ For these individuals, prediction accuracy is rather poor, especially for venous concentrations, and so application these results should be done with caution. It seems likely that more data and a more complex arteriovenous model are required, especially for patients treated in the ICU. Because of these uncertainties, we suggest that TCI for patients receiving ECMO or being treated in the ICU should use the unadjusted population model with titration-to-effect to achieve clinically relevant drug effects.

### Modelling tolerance

To evaluate tolerance to remimazolam, we tested a competitive interaction model and found improved PD model fit for both MOAA/S and BIS. The mechanism predicts reduced remimazolam sensitivity over time, which has previously been observed and modelled for two benzodiazepines,^26^ and suggested for remimazolam.^27^

In the competitive interaction model, CNS7054 has no pharmacological activity but competes with remimazolam for receptor occupancy. This mechanism is thought to be unlikely because of CNS7054 receptor affinity 300 times lower than for remimazolam.^2^ We used this approach because we did not find another mechanistic model that captures the tolerance behaviour. The approach of an additional effect-site for remimazolam-induced changes in Ce50 would have an extremely slow time constant (in the order of hours) which is difficult to explain and lacks a mechanistic explanation of how sensitivity is changed. The model of Zhou and colleagues^12^ increases remimazolam Ce50 from cumulative dose but this has no mechanistic explanation and it is unclear how to handle multiple infusions over a long period. In our model, the metabolite concentration serves as a measure of remimazolam exposure associated with development of tolerance. The effect also dissipates over time, which seems likely. What is needed are (pre-)clinical experiments to distinguish the different hypotheses around tolerance development and competitive inhibition.

For the metabolite CNS7054, the Ca50 for competitive interaction is 8–12 times higher than the Ce50 for remimazolam drug effect, while CNS7054 CL is about 20 times lower. These differences suggest that tolerance will have little influence for sedation and short duration anaesthesia. However, long duration anaesthesia and the presence of end-stage renal disease will result in greater exposure and might make this mechanism more consequential. It is not clear whether this is related to the time-dependent change in remimazolam drug CL found for >24 h treatment in the ICU and for long (>10 h) infusions found by Zhou and colleagues.^12^

Current commercial TCI systems target effect-site or central compartment concentrations from which clinicians must infer expected drug effects. When using a remimazolam TCI system and stopping an infusion after long duration anaesthesia, the presence of tolerance will result in drug effects diminishing faster than expected from the predicted remimazolam concentrations alone. It might also be necessary for clinicians to increase target concentrations over the course of long duration anaesthesia to achieve constant drug effects until steady-state is reached. It is not clear to what degree this is clinically relevant. Our model predicts this tolerance phenomenon will be more prevalent in children than in older individuals, a testable hypothesis.

### Limitations

This study has a number of limitations. Prediction accuracy for venous samples was worse than for arterial samples suggesting that the arteriovenous model could be improved, as is especially evident for patients in the ICU. Use of the sequential method limits the CNS7054 samples that can be used for model development as it requires remimazolam samples in the same individual. Similarly, for MOAA/S and BIS determinations, both remimazolam and CNS7054 samples in the same individuals are required. Size scalars such as fat-free mass^39^^,^^40^ or body weight adjustments were not investigated because height is a predictor in the formulae but was not available for all individuals. This study handled concomitant opioids as a binary covariate, while it is obvious that the concentration of an opioid would be impactful, however, the current data are not sufficient to develop a combined remimazolam–opioid model. Use of a remimazolam–CNS7054 competitive interaction in our model was intended to capture the well-known tolerance behaviour to benzodiazepines, however this mechanism is not certain and other mechanisms should be explored. The model equations address competition between remimazolam effect-site and CNS7054 plasma concentrations, which is not thought to be mechanistic, however doing otherwise did not improve the model.

### Conclusions

We developed a PK–PD model predicting remimazolam and CNS7054 concentrations and MOAA/S and BIS values for a broad population. Simulations indicate which target concentrations are likely to result in adequate clinical effect in sedation and anaesthesia. Dosing from the simulations agrees with clinical studies, promoting confidence that the model might be useful in routine clinical practice across diverse populations. Prospective validation of the model is required to determine its safety and efficacy.

## Authors’ contributions

Study design: DJE, PC, TS

Data analysis: DJE

Writing the manuscript: all authors

## Funding

Funding from the department of Anesthesiology, 10.13039/501100001721University of Groningen, 10.13039/501100005075University Medical Center Groningen, and by an unrestricted grant from PAION, Aachen, Germany.

## Declarations of interest

DJE is an editorial board member of *Anesthesiology*. He has received travel support from Becton Dickinson (Eysins, Switzerland). DJE, PC, JPVdB, and JVK share the departmental interests of MMRFS. TS is an employee of Paion, the European marketing authorisation holder of remimazolam. MMRFS’ research group or department received (over the last 3 years) research grants and consultancy fees from Masimo (Irvine, CA, USA), Becton Dickinson (Eysins, Switzerland), Fresenius-Kabi (Bad Homburg, Germany), Paion (Aachen, Germany), Medcaptain Europe (Andelst, The Netherlands), Baxter (Chicago, IL, USA), HanaPharm (Seoul, Republic of Korea). He receives royalties on intellectual property from Demed Medical (Sinaai, Belgium) and Ghent University (Ghent, Belgium).
