# Supplementary material for: Development and analysis of a remimazolam pharmacokinetics and pharmacodynamics model with proposed dosing and concentrations for anaesthesia and sedation
Source: Br J Anaesth. 2025 Apr 30;135(1):206–17. doi: 10.1016/j.bja.2025.02.038 (PMC12597572; doi:10.1016/j.bja.2025.02.038)
Supplement: Multimedia component 1 [file mmc1.docx]

## Supplementary content

### NONMEM code for $PK block

OA1P2=2 ; opiates present

P1GT8=1 ; normal hepatic function

H1ESRD2=1 ; normal renal function

AVFLAG=3 ; arterial and venous samples

; moaas

ME50M=EXP(THETA(39)+ETA(14)) )) ; metabolite CE50 for MOAAS

PE50=EXP(THETA(38)+ETA(13)+THETA(40)/1000*(AGE-35)) ; remi CE50 for MOAAS

DEFF=EXP(THETA(31)+ETA(12)) ; drug effect multiplier

MKE0=EXP(THETA(32)) ; remi ke0 for MOAAS

; bis

ME50B=EXP(THETA(45)) ; metabolite CE50 for BIS

BE50 =EXP(THETA(41)+ETA(13)+THETA(46)/1000*(AGE-35)) ; remi CE50 for BIS

BKE0 =EXP(THETA(42)+ETA(14)+THETA(47)/1000*(AGE-35)) ; remi ke0 for BIS

BISSD=EXP(THETA(44)) ; BIS additive error

BASE =THETA(43)+BISSD*ETA(15) ; BIS baseline

; metabolite PK

KAQ2=THETA(30)/1000*(AGE-35) ; metabolite AGE on Q2

KPQ2=THETA(29)/100*(P1GT8-1) ; metabolite hepatic function on Q2

KMOCL=THETA(28)/100*(OA1P2-1) ; metabolite opiates on CL

VSCA=(WGT/70)**THETA(27) ; metabolite weight scaling

KRCL=THETA(26)/100*(H1ESRD2-1) ; metabolite renal function on CL

V1M=EXP(THETA(19)+ETA(7))*VSCA ; metabolite V1

CLM=EXP(THETA(20)+ETA(8)+KRCL+KMOCL)*(VSCA**0.75) ; metabolite CL

KMO=CLM/V1M

KMD=EXP(THETA(21)+ETA(9))*(VSCA**(-0.25)) ; metabolite depot rate

MARTSD=EXP(THETA(22)) ; metabolite arterial error

MVENSD=EXP(THETA(23)) ; metabolite venous error

V2M=EXP(THETA(24)+ETA(10))*VSCA ; metabolite V2

Q2M=EXP(THETA(25)+ETA(11)+KPQ2+KAQ2)*((V2M/EXP(THETA(24)))**0.75); met Q2

K21M=Q2M/V2M

K12M=Q2M/V1M

MRAT=425.3/439.3 * 1.0 ; Molecular weight correction and 100% conversion

; remimazolam PK

ETCLV=1

IF (AVFLAG.EQ.2) ETCLV=THETA(16) ; remi CL var for venous

KHV3=THETA(15)/100*(P1GT8-1) ; remi hepatic function on V3

KOCL=THETA(14)/100*(OA1P2-1) ; remi opiates on CL

KSV3=THETA(13)/100*(M1F2-1) ; remi sex on V3

KSCL=THETA(12)/100*(M1F2-1) ; remi sex on CL

KAV3=THETA(11)/1000*(AGE-35) ; remi age on V3

; classical venous model

VKE0=EXP(THETA(10))*((WGT/70)**(-0.25)) ; venous delay

VSIZ=WGT/70

CSIZ=(WGT/70)**0.75

V1=VSIZ*EXP(THETA(1)+ETA(1)) ; remi V1

V2=VSIZ*EXP(THETA(2)+ETA(2)) ; remi V2

V3=VSIZ*EXP(THETA(3)+ETA(3)+KAV3+KSV3+KHV3) ; remi V3

CL=CSIZ*EXP(THETA(4)+ETA(4)*ETCLV+KSCL+KOCL) ; remi CL

Q2=((V2/EXP(THETA(2)))**0.75)*EXP(THETA(5)+ETA(5)) ; remi Q2

Q3=((V3/EXP(THETA(3)))**0.75)*EXP(THETA(6)+ETA(6)) ; remi Q3

ARTSD=EXP(THETA(7))

S1=V1

VAFRA=EXP(THETA(9)) ; remi arterial error

VENSD=EXP(THETA(8)) ; remi venous error

K10=CL/V1

K12=Q2/V1

K21=Q2/V2

K13=Q3/V1

K31=Q3/V3

K123=K10+K12+K13

### NONMEM code for $DES

DADT(1) = A(2)*K21 + A(3)*K31 - A(1)*K123 ; remimazolam central

DADT(2) = A(1)*K12 - A(2)*K21 ; remimazolam peripheral 1

DADT(3) = A(1)*K13 - A(3)*K31 ; remimazolam peripheral 2

DADT(4) = VKE0*(A(1)/V1 - A(4)) ; remimazolam venous delay

DADT(5) = A(1)*K10*MRAT - A(5)*KMD ; depot

KKM2=A(6)*K12M - A(7)*K21M

DADT(6) = A(5)*KMD - A(6)*KMO - KKM2 ; CNS7054 central

DADT(7) = KKM2 ; CNS7054 peripheral

DADT(8) = VKE0*(A(6)/V1M - A(8)) ; CNS7054 venous delay

DADT(9) = MKE0*(A(1)/V1 - A(9)) ; MOAAS effect site

DADT(10) = BKE0*(A(1)/V1 - A(10)) ; effect site

### NONMEM code for $THETA

(1.460310e+00 FIXED) ; remimazolam log(v1)

(2.509550e+00 FIXED) ; remimazolam log(v2)

(2.925370e+00 FIXED) ; remimazolam log(v3)

(1.131190e-01 FIXED) ; remimazolam log(cl)

(3.733570e-01 FIXED) ; remimazolam log(q2)

(-1.211240e+00 FIXED) ; remimazolam log(q3)

(-2.115340e+00 FIXED) ; remimazolam arterial log(SD)

(-9.761850e-01 FIXED) ; remimazolam venous log(SD)

(-2.022110e+00 FIXED) ; venous log(fraction)

(-4.237270e+00 FIXED) ; venous log(ke0)

(7.308170e+00 FIXED) ; remimazolam age~v3

(1.628020e+01 FIXED) ; remimazolam sex~cl

(2.870370e+01 FIXED) ; remimazolam sex~v3

(-1.393400e+01 FIXED) ; remimazolam opiates~cl

(8.237880e+01 FIXED) ; remimazolam hepatic~v3

(1.861520e+00 FIXED) ; venous variance CL

(0 FIXED)

(0 FIXED)

(1.924610e+00 FIXED) ; CNS7054 log(v1)

(-2.710040e+00 FIXED) ; CNS7054 log(cl)

(-1.535490e+00 FIXED) ; CNS7054 log(kdepot)

(-2.730940e+00 FIXED) ; CNS7054 arterial log(SD)

(-1.810620e+00 FIXED) ; CNS7054 venous log(SD)

(1.633100e+00 FIXED) ; CNS7054 log(v2)

(-1.961730e+00 FIXED) ; CNS7054 log(q2)

(-2.182610e+02 FIXED) ; CNS7054 renal~cl

(5.184820e-01 FIXED) ; CNS7054 size power

(-3.275030e+01 FIXED) ; CNS7054 opiates~cl

(1.882800e+02 FIXED) ; CNS7054 hepatic~q2

(1.574090e+01 FIXED) ; CNS7054 age~q2

(2.990080e+00 FIXED) ; MOAAS drug effect scale

(-1.209760e+00 FIXED) ; keoref=0.2983

(-1.659520e+01 FIXED) ; odds p0

(9.756720e-01 FIXED) ; odds p1 2.65

(-5.955830e-02 FIXED) ; odds p2 0.94

(3.851300e-01 FIXED) ; odds p3 1.47

(9.320940e-01 FIXED) ; odds p4 2.54

(-1.703500e+00 FIXED) ; pe50=0.182

(2.478340e+00 FIXED) ; me50=11.921

(-7.626000e+00 FIXED) ; age~pe50

(-1.780770e-02 FIXED) ; e50(bis)=0.982350

(-1.931140e+00 FIXED) ; keoref=0.1450

(9.371960e+01 FIXED) ; baseline

(1.914410e+00 FIXED) ; BIS error=6.782936

(2.129380e+00 FIXED) ; e50(met)=8.409651

(-1.640380e+01 FIXED) ; age e50

(-1.064560e+01 FIXED) ; age ke0

(1.668420e+00 FIXED) ; MOAAS 0 opioids offset

### NONMEM code for $ERR

APRED = A(1)/V1 ; remimazolam arterial

VPRED = VAFRA*A(4) + (1-VAFRA)*APRED ; remimazolam venous

MAPRED = A(6)/V1M ; CNS7054 arterial

MVPRED = VAFRA*A(8) + (1-VAFRA)*MAPRED ; CNS7054 venous

CMET=MAPRED/1000

IF (CMET.LT.0) CMET=0

CMETRAT=CMET/ME50M

; bis

CEFFB=A(10)/1000

IF (CEFFB.LT.0) CEFFB=0

; moaas

CEFFM=A(9)/1000

IF (CEFFM.LT.0) CEFFM=0

; BIS model

PEFFB=(CEFFB/BE50)/(1 + CEFFB/BE50 + CMET/ME50B)

BPRED=BASE - BASE*PEFFB

; MOAA/S model

PEFFM=DEFF*(CEFFM/PE50)/(1 + CEFFM/PE50 + CMET/ME50M)

;baseline values

DEOP=THETA(48)*(OA1P2-1)

DEB0=EXP(THETA(34))-EXP(THETA(34)-DEOP)

B0 =THETA(33)+DEB0 ; logit Pr[Y<=0]

D01=EXP(THETA(34)-DEOP) ; Difference between logit Pr[Y<=0] and Pr[Y<=1]

D12=EXP(THETA(35))

D23=EXP(THETA(36))

D34=EXP(THETA(37))

;logits for Pr[Y<=0], Pr[Y<=1], etc...

LLE0=B0+PEFFM

LLE1=LLE0+D01

LLE2=LLE1+D12

LLE3=LLE2+D23

LLE4=LLE3+D34

;Pr[Y<=0], Pr[Y<=1], etc...

PLE0=EXP(LLE0)/(1+EXP(LLE0))

PLE1=EXP(LLE1)/(1+EXP(LLE1))

PLE2=EXP(LLE2)/(1+EXP(LLE2))

PLE3=EXP(LLE3)/(1+EXP(LLE3))

PLE4=EXP(LLE4)/(1+EXP(LLE4))

;Pr[Y=0], Pr[Y=1], etc...

P5=1-PLE4

P4=PLE4-PLE3

P3=PLE3-PLE2

P2=PLE2-PLE1

P1=PLE1-PLE0

P0=PLE0

IF (P5.LT.0) P5=0

IF (P4.LT.0) P4=0

IF (P3.LT.0) P3=0

IF (P2.LT.0) P2=0

IF (P1.LT.0) P1=0

IF (P0.LT.0) P0=0

;probability weighted MOAAS

MPRED=0*P0+1*P1+2*P2+3*P3+4*P4+5*P5

## Supplementary Tables

Supplementary Table 1: The studies included for analysis

| **Study** | **Individuals** | **Sessions** | | | | **Reference** |
| --- | --- | --- | --- | --- | --- | --- |
|  | All data | Remimazolam | CNS7054 | MOAA/S | BIS |  |
| CNS7056-001 | 54 | 54 | 54 | 54 | 54 | Antonik LJ, Goldwater DR, Kilpatrick GJ, Tilbrook GS, Borkett KM. A placebo-and midazolam-controlled phase I single ascending-dose study evaluating the safety, pharmacokinetics, and pharmacodynamics of remimazolam (CNS 7056): Part I. Safety, efficacy, and basic pharmacokinetics. *Anesth Analg* 2012; **115**: 274-83  Wiltshire HR, Kilpatrick GJ, Tilbrook GS, Borkett KM. A placebo-and midazolam controlled phase I single ascending-dose study evaluating the safety, pharmacokinetics, and pharmacodynamics of remimazolam (CNS 7056): part II. Population pharmacokinetic and pharmacodynamic modeling and simulation. *Anesth Analg* 2012; **115**: 284–96 |
| CNS7056-002 | 45 | 45 | 45 | 45 | 45 | Worthington MT, Antonik LJ, Goldwater DR, et al. A phase Ib, dose-finding study of multiple doses of remimazolam (CNS 7056) in volunteers undergoing colonoscopy. *Anesth Analg* 2013; **117**: 1093-100 |
| CNS7056-004 | 119 | 28 | 28 | 28 |  | Pambianco DJ, Borkett KM, Riff DS, et al. A phase IIb study comparing the safety and efficacy of remimazolam and midazolam in patients undergoing colonoscopy. *Gastrointest Endosc* 2016; **83**: 984-92 |
| CNS7056-006 | 296 | 85 | 83 | 83 |  | Rex DK, Bhandari R, Desta T, et al. A phase III study evaluating the efficacy and safety of remimazolam (CNS 7056) compared with placebo and midazolam in patients undergoing colonoscopy. *Gastrointest Endosc* 2018; **88**: 427-437 |
| CNS7056-008 | 303 | 4 | 4 | 4 |  | Pastis NJ, Yarmus LB, Schippers F, et al. Safety and efficacy of remimazolam compared with placebo and midazolam for moderate sedation during bronchoscopy. *Chest* 2019; **155**: 137-46 |
| CNS7056-010 | 62 | Posthoc PK analysis  Extracorporeal Membrane Oxygenation (ECMO) | | | | Bevilacqua C, Probst S, Soehngen M, Ender J. The sedative effect of remimazolam in general anesthesia for cardiac surgery measured by Narcotrend. *J Cardiothorac Vasc Anesth* 2015; **29**: S50-S1 |
| CNS7056-012 | 19 | 19 | 18 |  |  | Stöhr T, Colin PJ, Ossig J, et al. Pharmacokinetic properties of remimazolam in subjects with hepatic or renal impairment. *Br J Anaesth* 2021; **127**: 415–23 |
| CNS7056-015 | 31 | 31 | 29 | 29 |  | Rex DK, Bhandari R, Lorch DG, et al. Safety and efficacy of remimazolam in high risk colonoscopy: a randomized trial. *Dig Liver Dis* 2021; **53**: 94-101 |
| CNS7056-017 | 20 | 20 | 20 | 20 |  | Kleiman RB, Darpo B, Thorn M, Stoehr T, Schippers F. Potential strategy for assessing QT/QTc interval for drugs that produce rapid changes in heart rate: Electrocardiographic assessment of the effects of intravenous remimazolam on cardiac repolarization. *Br J Clin Pharmacol* 2020; **86**: 1600-9 |
| CNS7056-022 | 289 | 82 | 82 | 72 |  | Fechner J, El‐Boghdadly K, Spahn DR, et al. Anaesthetic efficacy and postinduction hypotension with remimazolam compared with propofol: a multicentre randomised controlled trial. *Anaesthesia* 2024; **79**: 410-422 |
| CNS7056-025 | 24 | 72 | 72 | 72 | 72 | Vellinga R, Koomen JV, Eleveld DJ, et al. Target-controlled Infusion of Remimazolam in Healthy Volunteers Shows Some Acute Tolerance. *Anesthesiology* 2024; **140**: 207-19 |
| CNS7056-026 | 31 | 28 | 28 |  |  | MMRF Struys. 2023. Pharmacokinetics and Pharmacodynamics of Remimazolam for Procedural Sedation in Children and Adolescents. ISAP San Francisco, California, USA |
| ONO-2745-01 | 35 | 35 | 20 | 20 | 20 | Doi M. Remimazolam. *J Jpn Soc Clin Anesth* 2014; **34**: 860-6 |
| ONO-2745-02 | 8 | 8 |  |  |  | Doi M. Remimazolam. *J Jpn Soc Clin Anesth* 2014; **34**: 860-6 |
| ONO-2745-03 | 85 | 40 | 40 |  | 40 | Doi M. Remimazolam. *J Jpn Soc Clin Anesth* 2014; **34**: 860-6 |
| ONO-2745-04 | 49 | Posthoc PK analysis  Treatment in the intensive care unit | | | | Petersen KU. Pre-clinical profile of Remimazolam and pharmacokinetics. 3rd annual meeting of the Japan Society for Clinical *Anesthesia*. 2017. Tokyo, Japan |
| ONO-2745-05 | 300 | 300 | 300 |  | 300 | Doi M, Hirata N, Suzuki T, et al. Safety and efficacy of remimazolam in induction and maintenance of general anesthesia in high-risk surgical patients (ASA Class III): results of a multicenter, randomized, double-blind, parallel-group comparative trial. *J Anesth* 2020; **34**: 491-501 |
| ONO-2745-06 | 62 | 62 | 62 |  | 62 | Doi M, Morita K, Takeda J, et al. Efficacy and safety of remimazolam versus propofol for general anesthesia: a multicenter, single-blind, randomized, parallel-group, phase IIb/III trial. *J Anesth* 2020; **34**: 543–53 |
| ONO-2745ivu007 | 20 | 20 | 20 | 20 | 20 | Stöhr T, Colin PJ, Ossig J, et al. Pharmacokinetic properties of remimazolam in subjects with hepatic or renal impairment. *Br J Anaesth* 2021; **127**: 415–23 |
| REHSCU_ICU | 30 | Posthoc PK analysis  Treatment in the intensive care unit | | | | Unpublished (data on file, PAION, Aachen, Germany) |

Supplementary Table 2: Modified Observer's Assessment of Alertness and Sedation (MOAA/S) score

| **MOAA/S score** | **Sedation depth** |  |
| --- | --- | --- |
| 5 | None | Responds readily to name spoken in normal tone |
| 4 | Minimal | Lethargic response to name spoken in normal tone |
| 3 | Moderate | Responds only after name is called loudly and/or repeatedly |
| 2 | Moderate | Responds only after mild prodding or shaking |
| 1 | Moderate | Responds only after painful trapezius squeeze |
| 0 | Deep | Does not respond to painful trapezius squeeze |

## Supplementary Figures


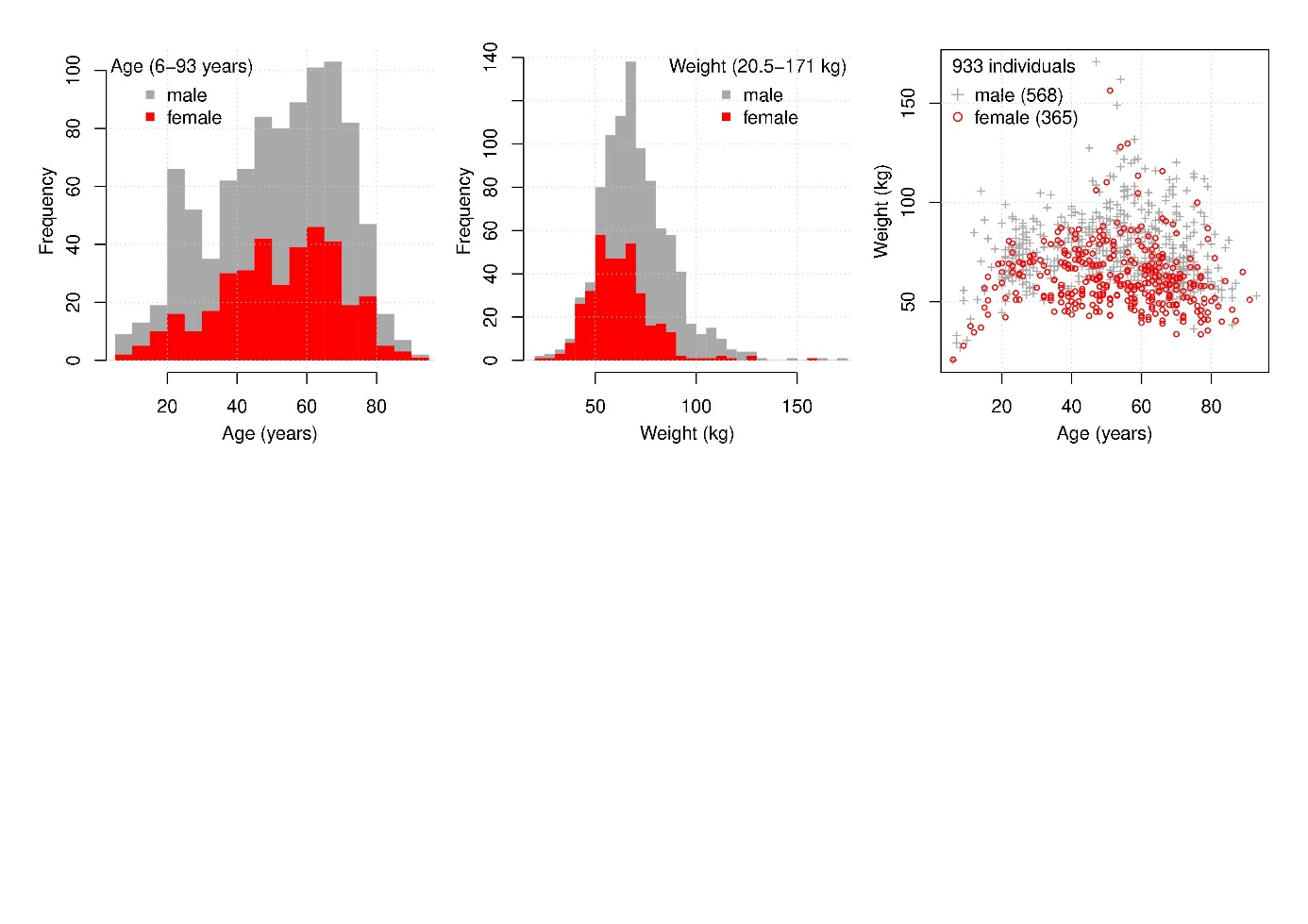


Supplementary Figure 1: Demographic information for individuals used for PK model development.

**
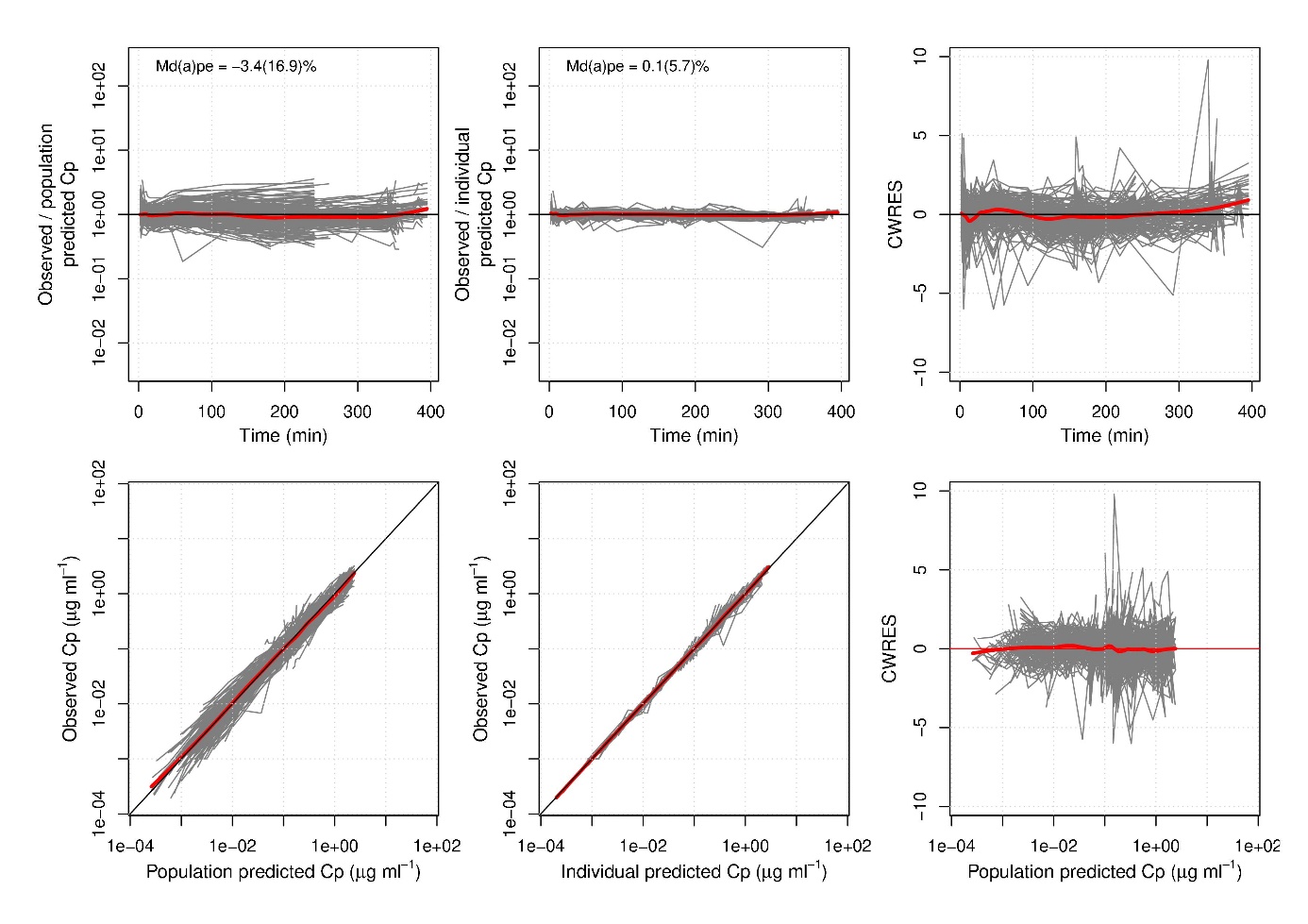
**

Supplementary Figure 2: Diagnostic plots for the remimazolam PK model to predict arterial samples. The red line shows a LOESS smoother.


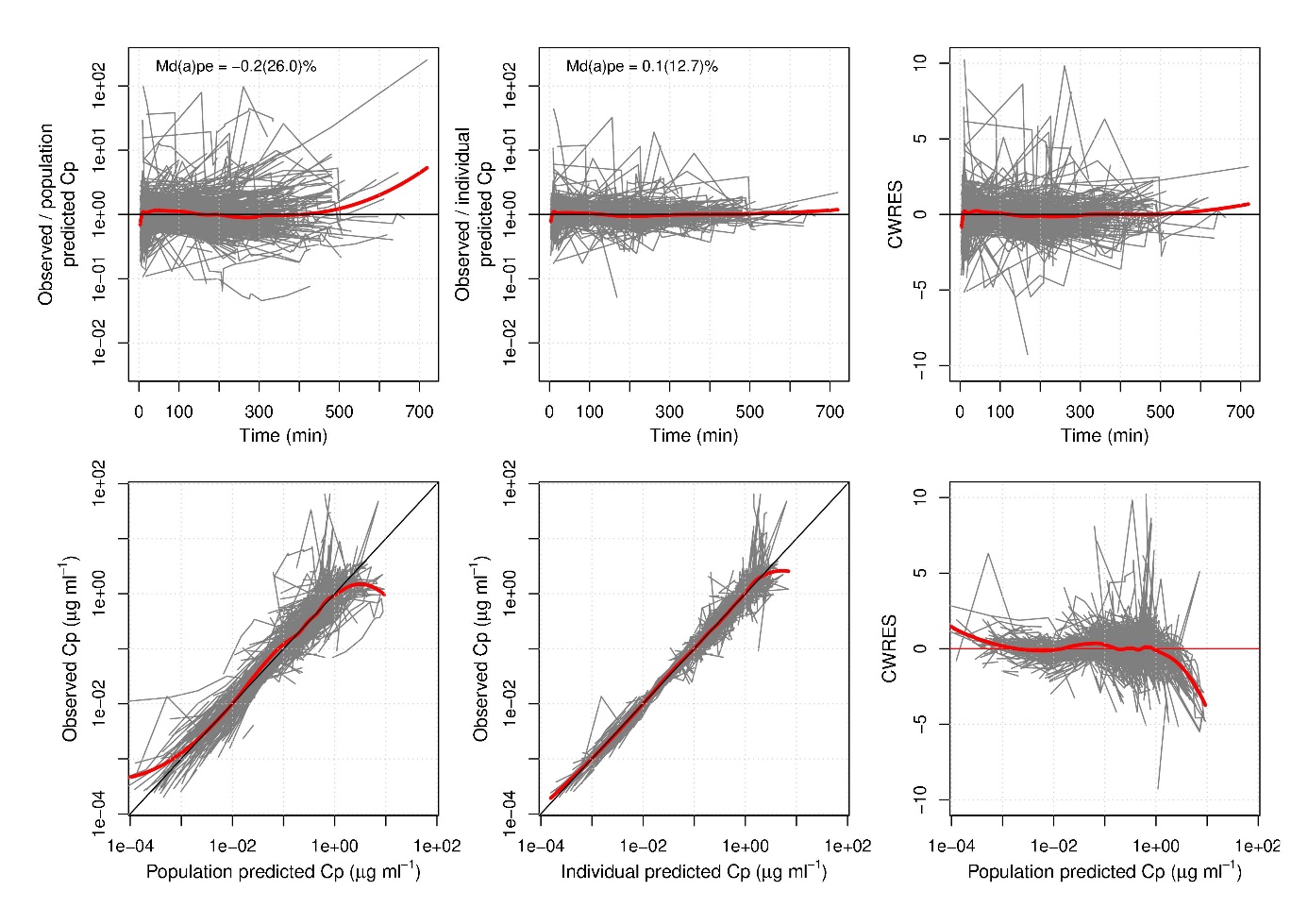


Supplementary Figure 3: Diagnostic plots for the remimazolam PK model to predict venous samples. The red line shows a LOESS smoother.


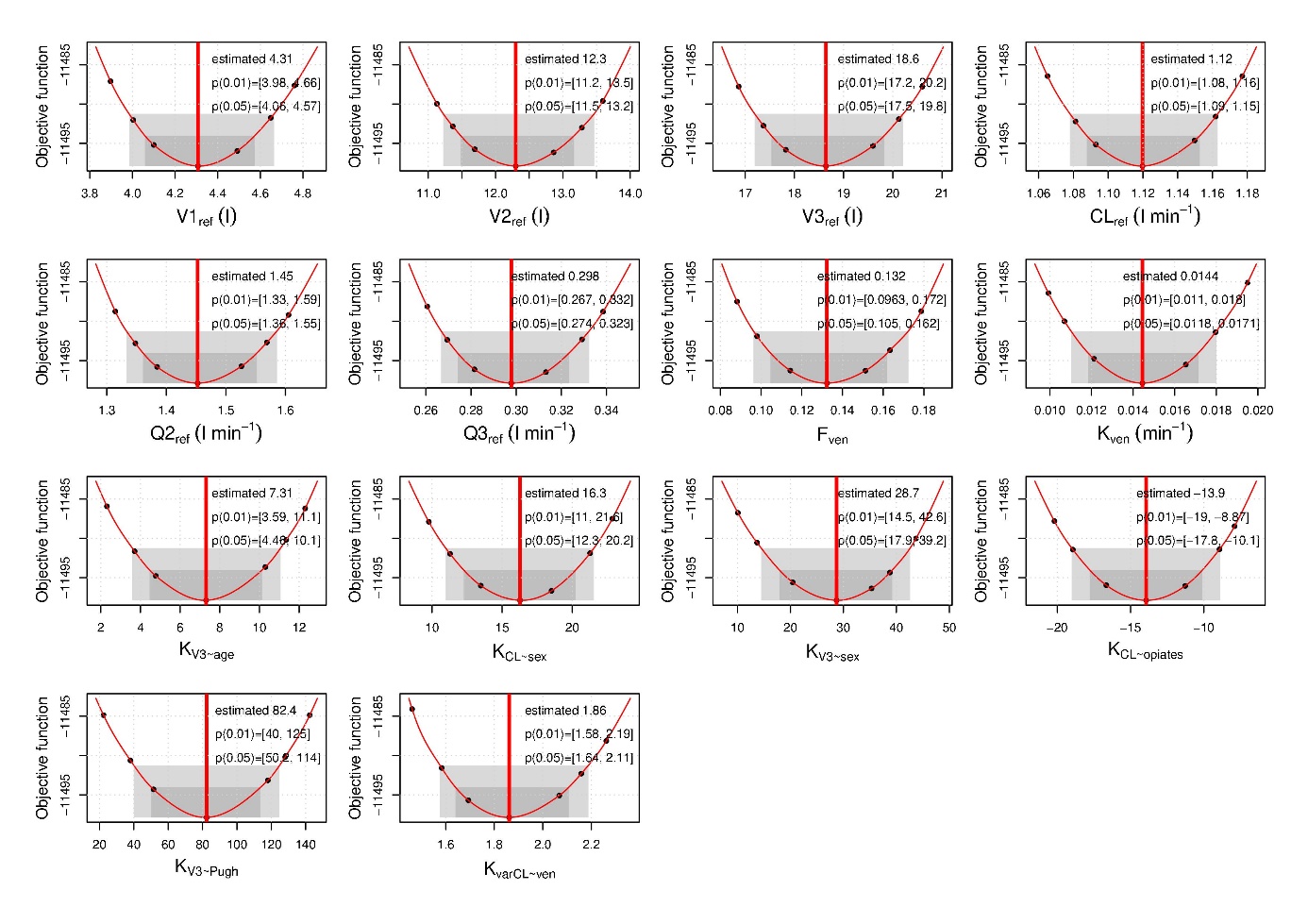


Supplementary Figure 4: Likelihood profiles for the remimazolam PK model.


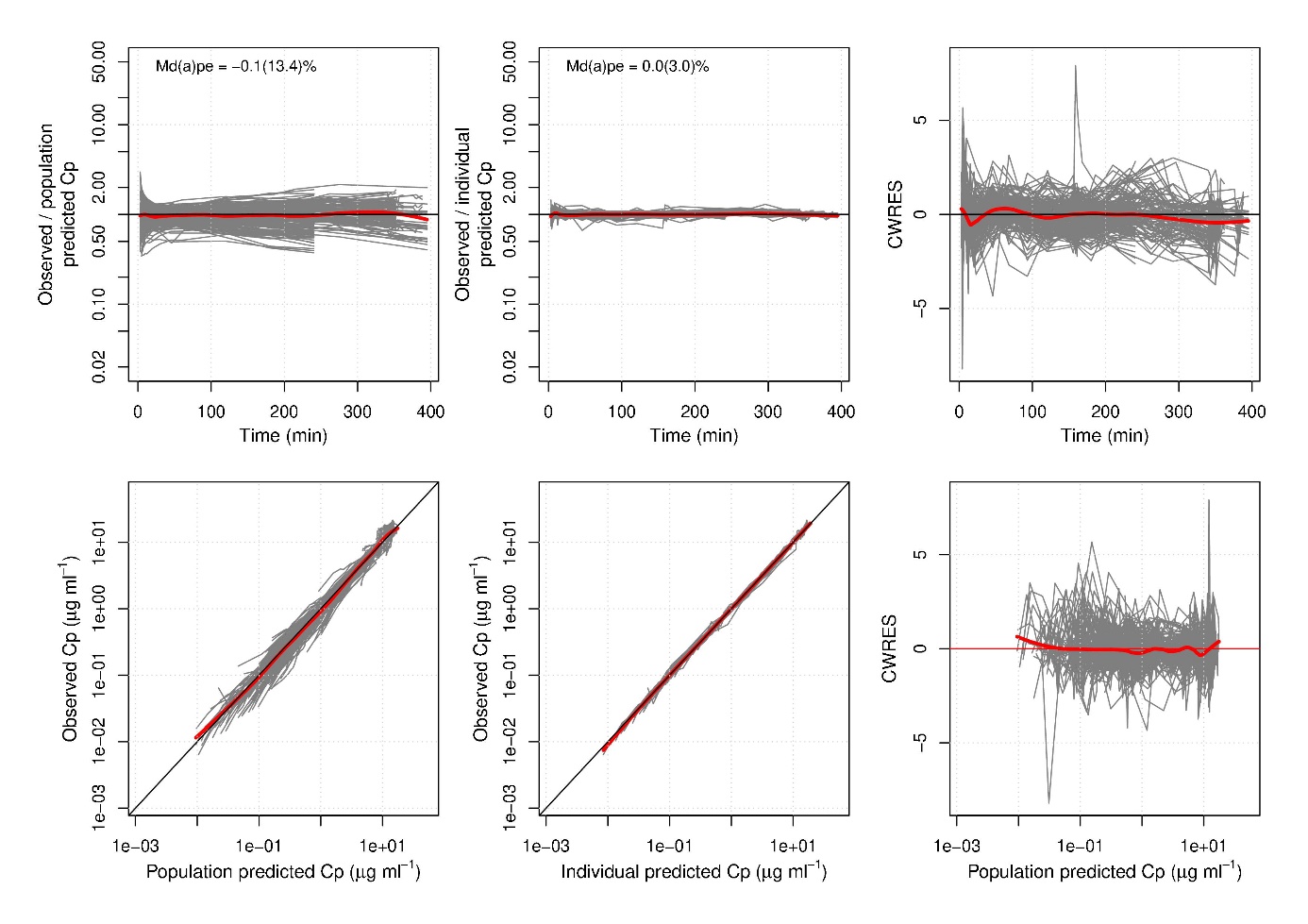


Supplementary Figure 5: Diagnostic plots for the CNS7054 PK model to predict arterial samples. The red line shows a LOESS smoother.


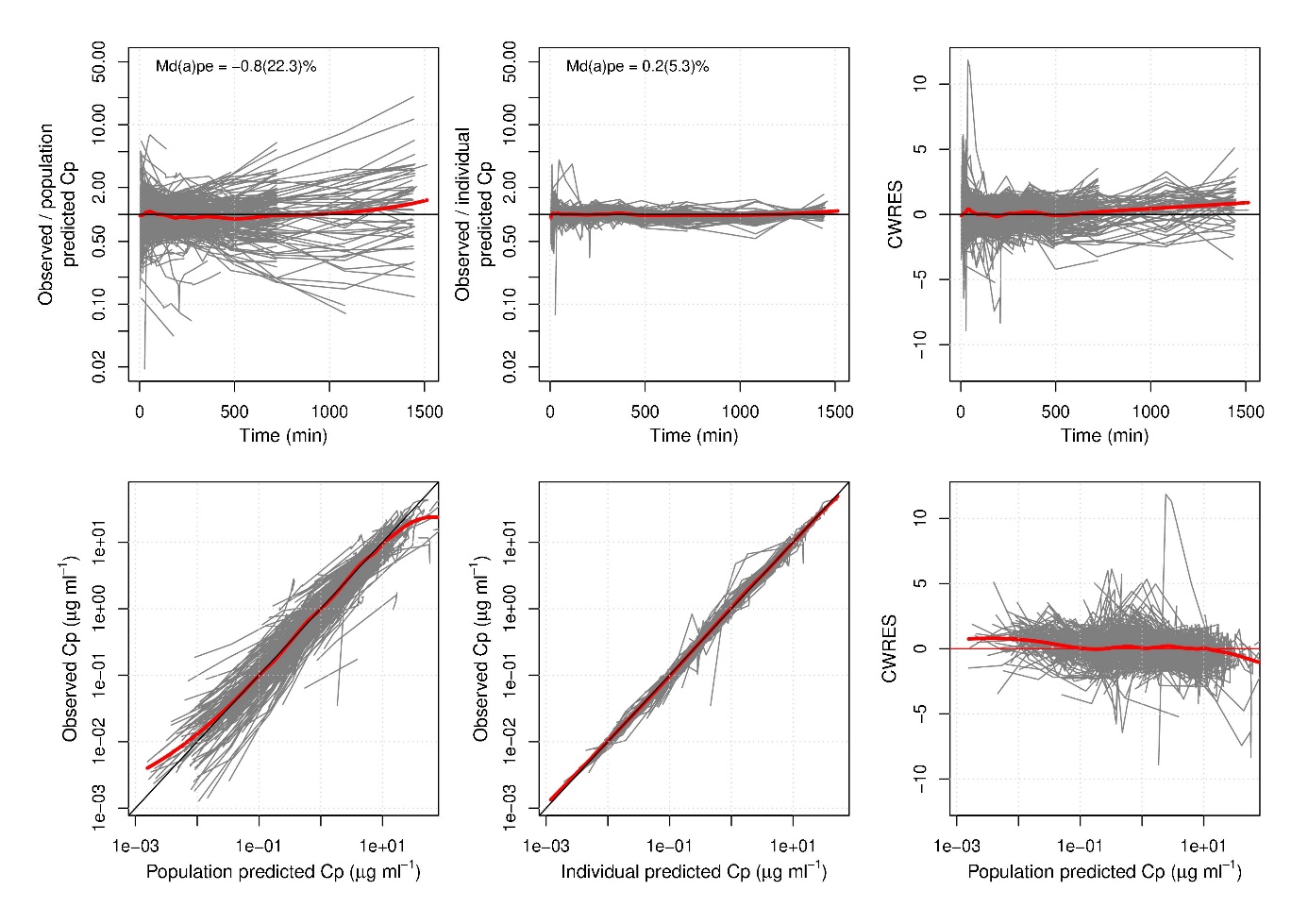


Supplementary Figure 6: Diagnostic plots for the CNS7054 PK model to predict venous samples. The red line shows a LOESS smoother.


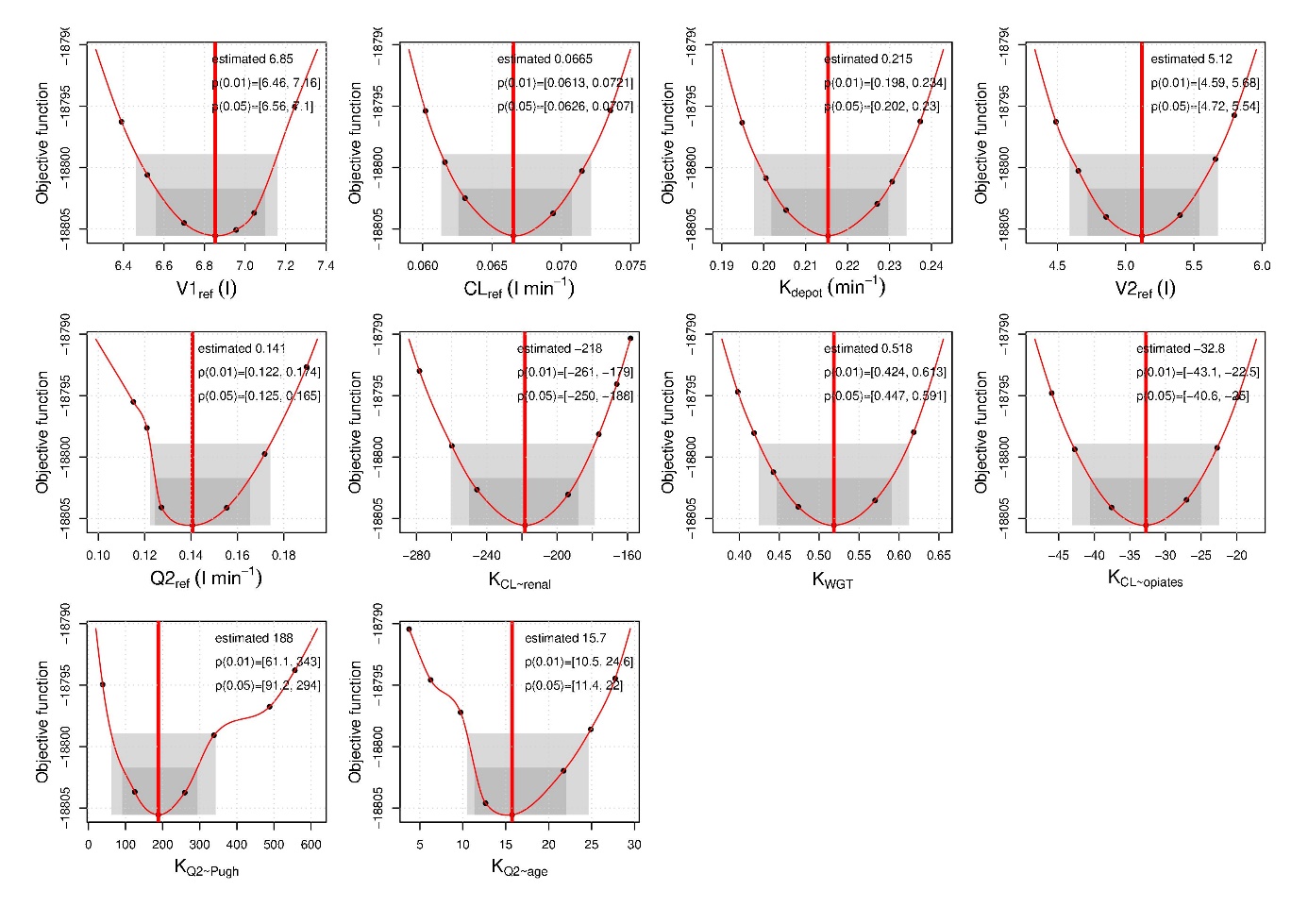


Supplementary Figure 7: Likelihood profiles for the CNS7054 PK model.


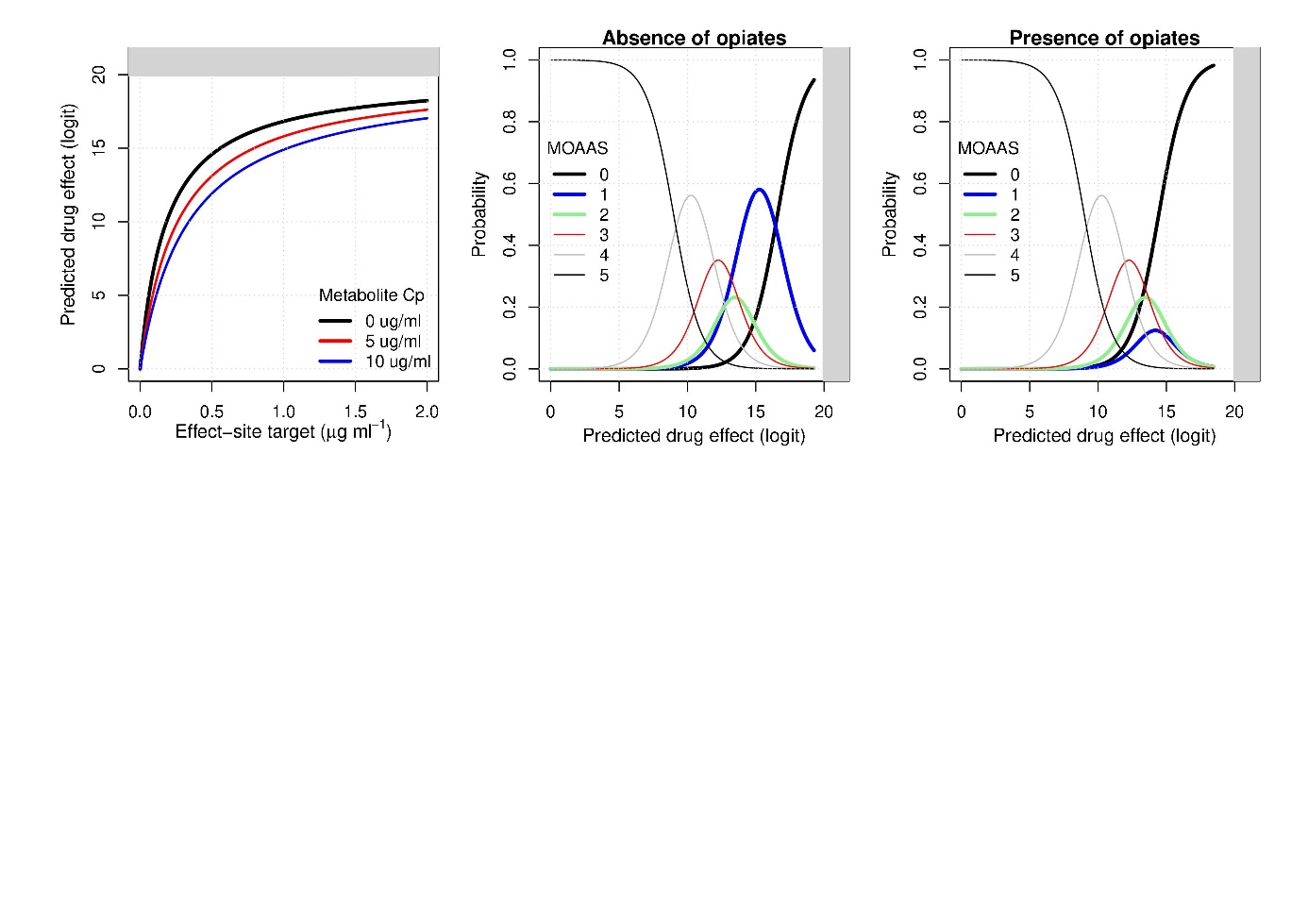


Supplementary Figure 8: The predicted relationship between effect-site concentration and MOAA/S score. The shaded area is unobtainable for any remimazolam concentration.


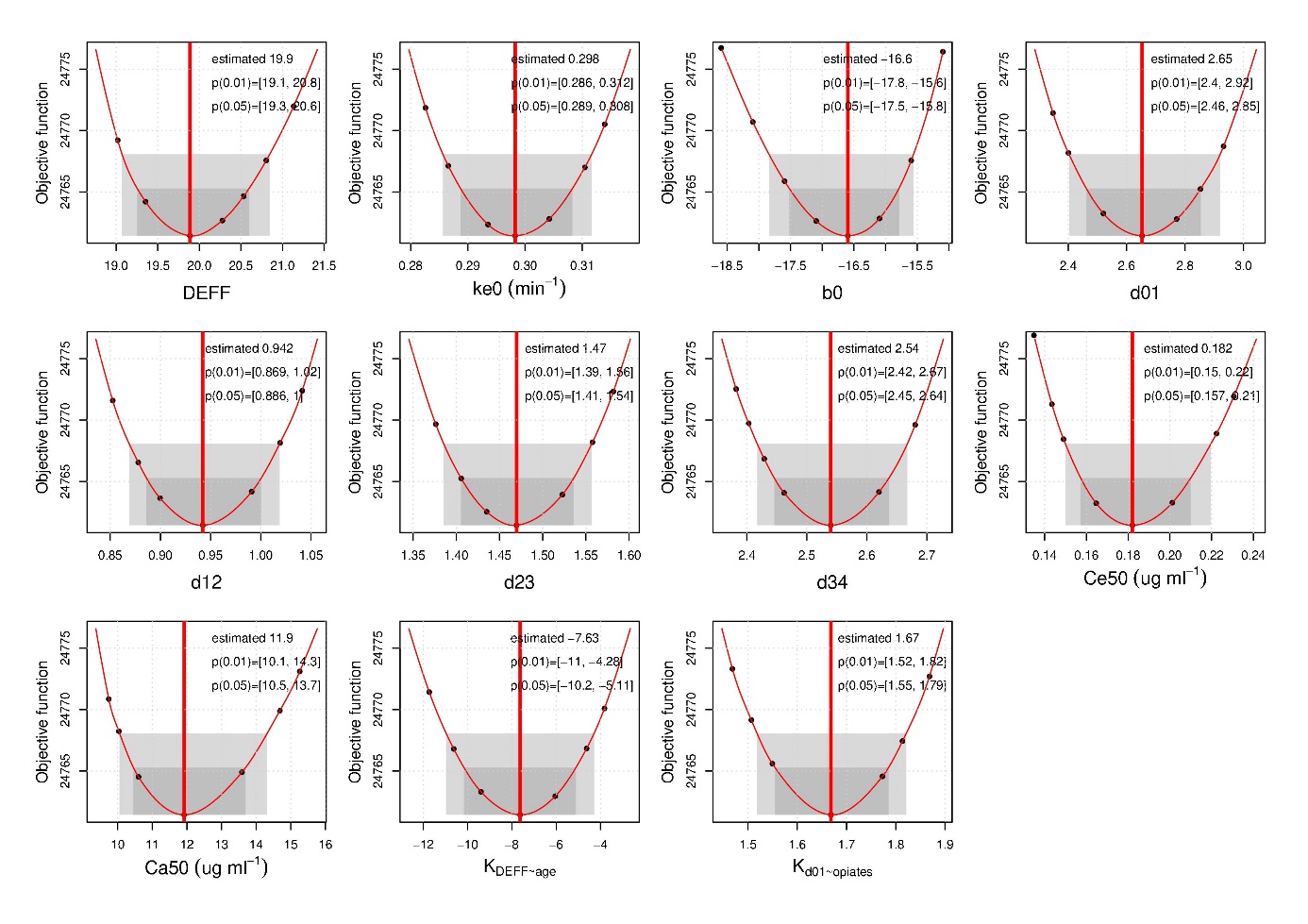


Supplementary Figure 9: Likelihood profiles for the MOAA/S PD model.


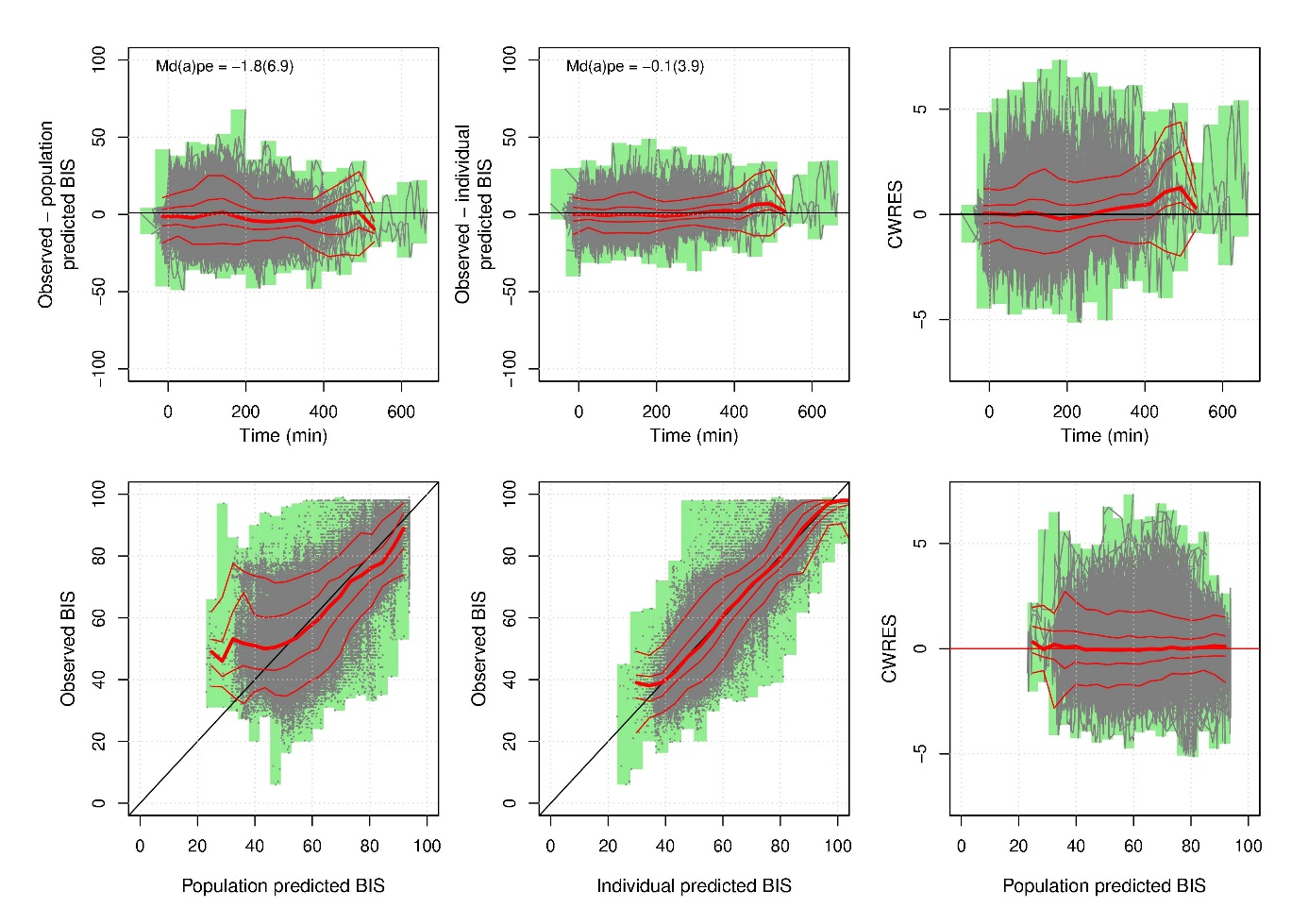


Supplementary Figure 10: Diagnostic plots for the BIS PD model. The red lines indicate the 5, 25, 50, 75, and 95 percentiles. The green shaded area indicates the maximum and minimum values.


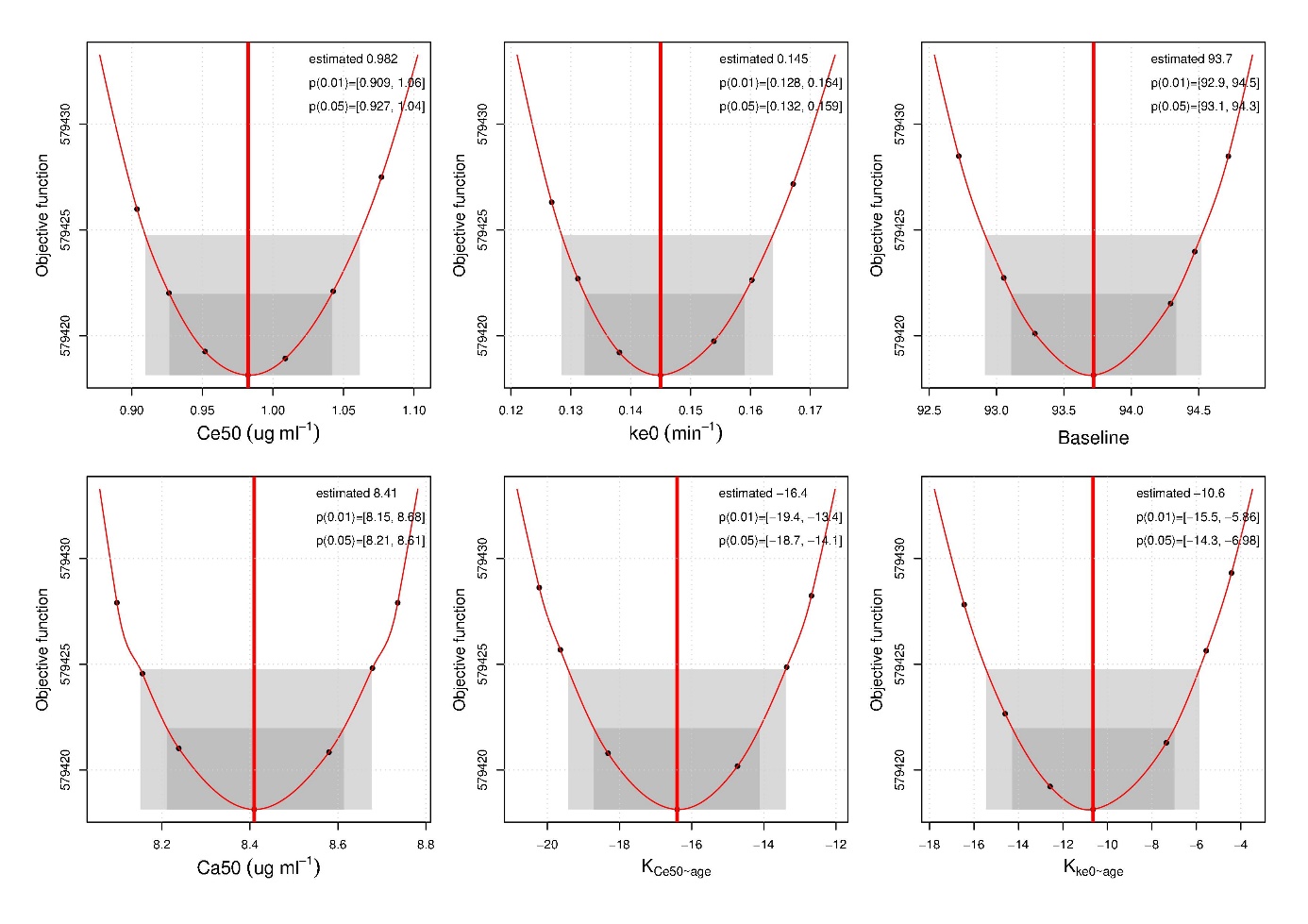


Supplementary Figure 11: Likelihood profiles for the BIS PD model.


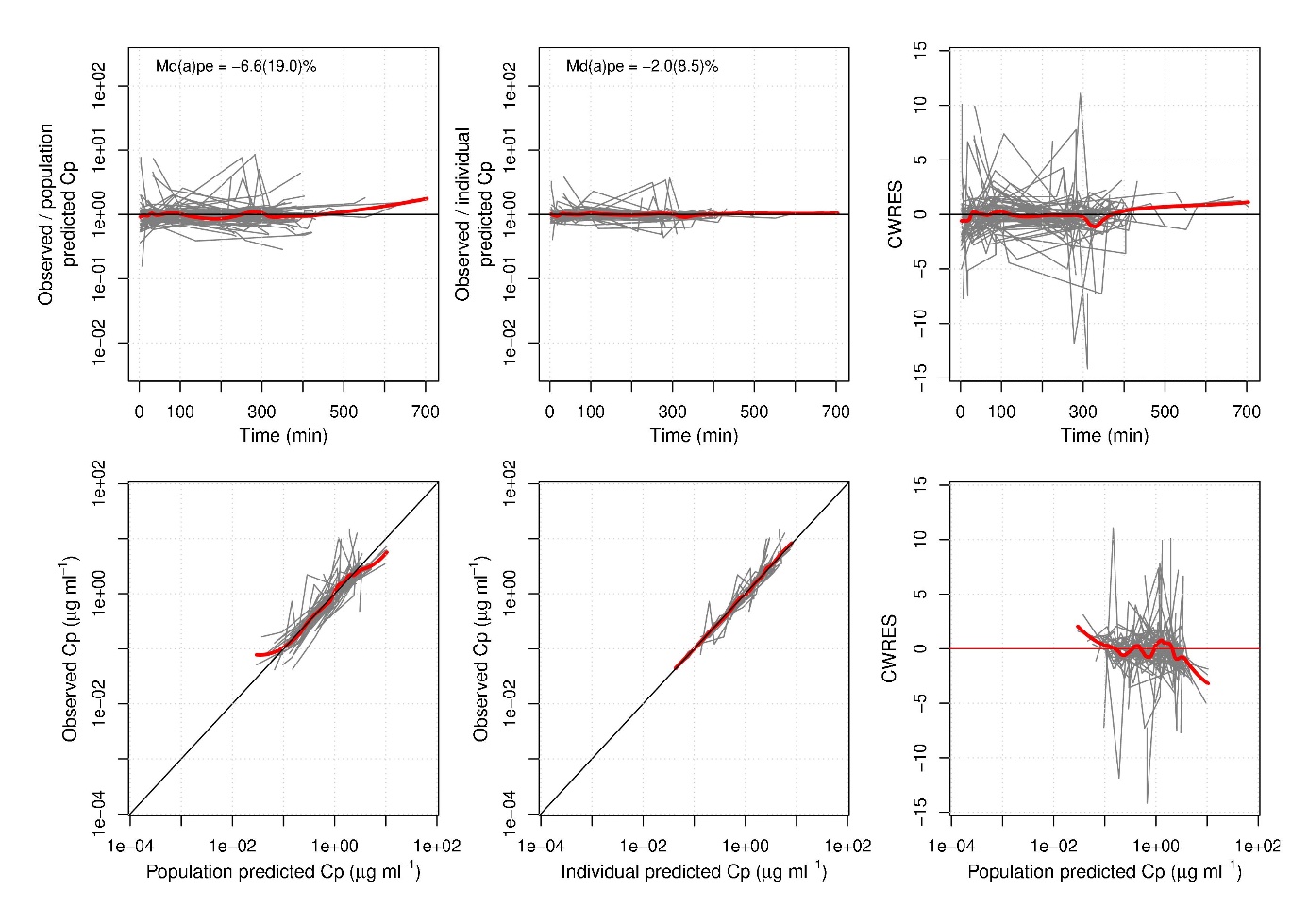


Supplementary Figure 12: Diagnostic plots for the adjusted posthoc PK model to predict remimazolam arterial samples for patients receiving ECMO. The red line shows a LOESS smoother.


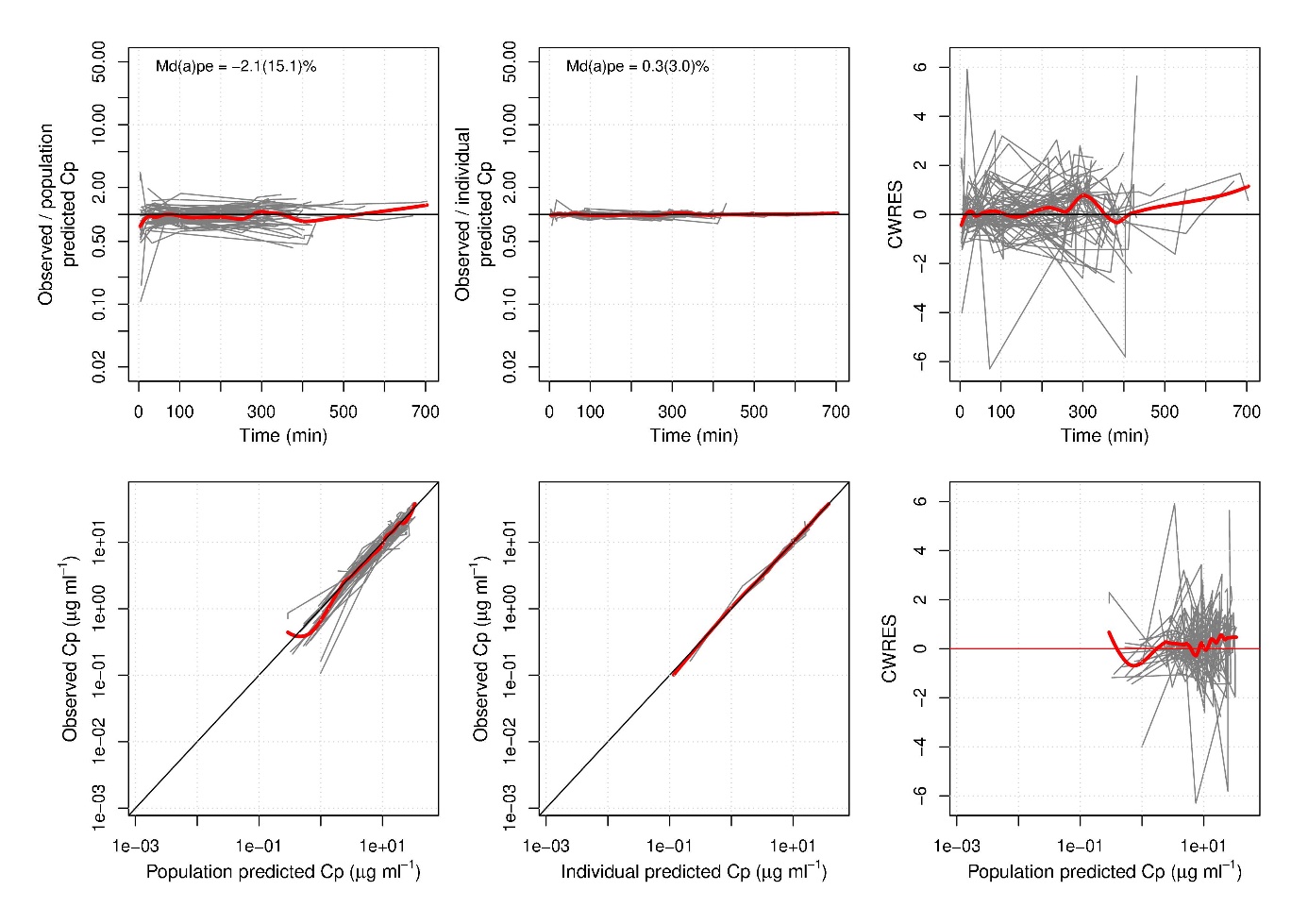


Supplementary Figure 13: Diagnostic plots for the adjusted posthoc PK model to predict CNS7054 arterial samples for patients receiving ECMO. The red line shows a LOESS smoother.


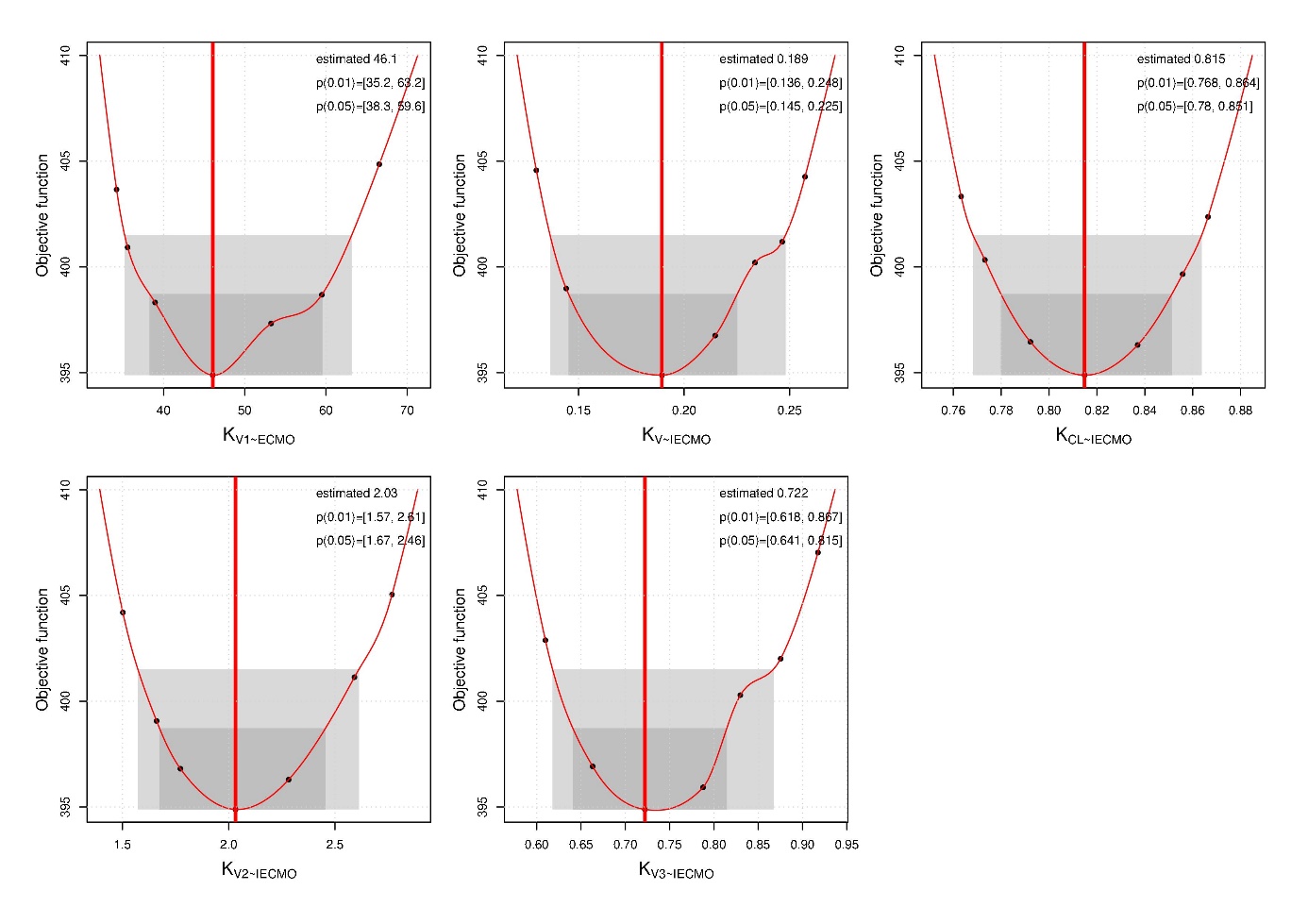


Supplementary Figure 14: Likelihood profiles for the PK model adjustments for patients receiving ECMO.


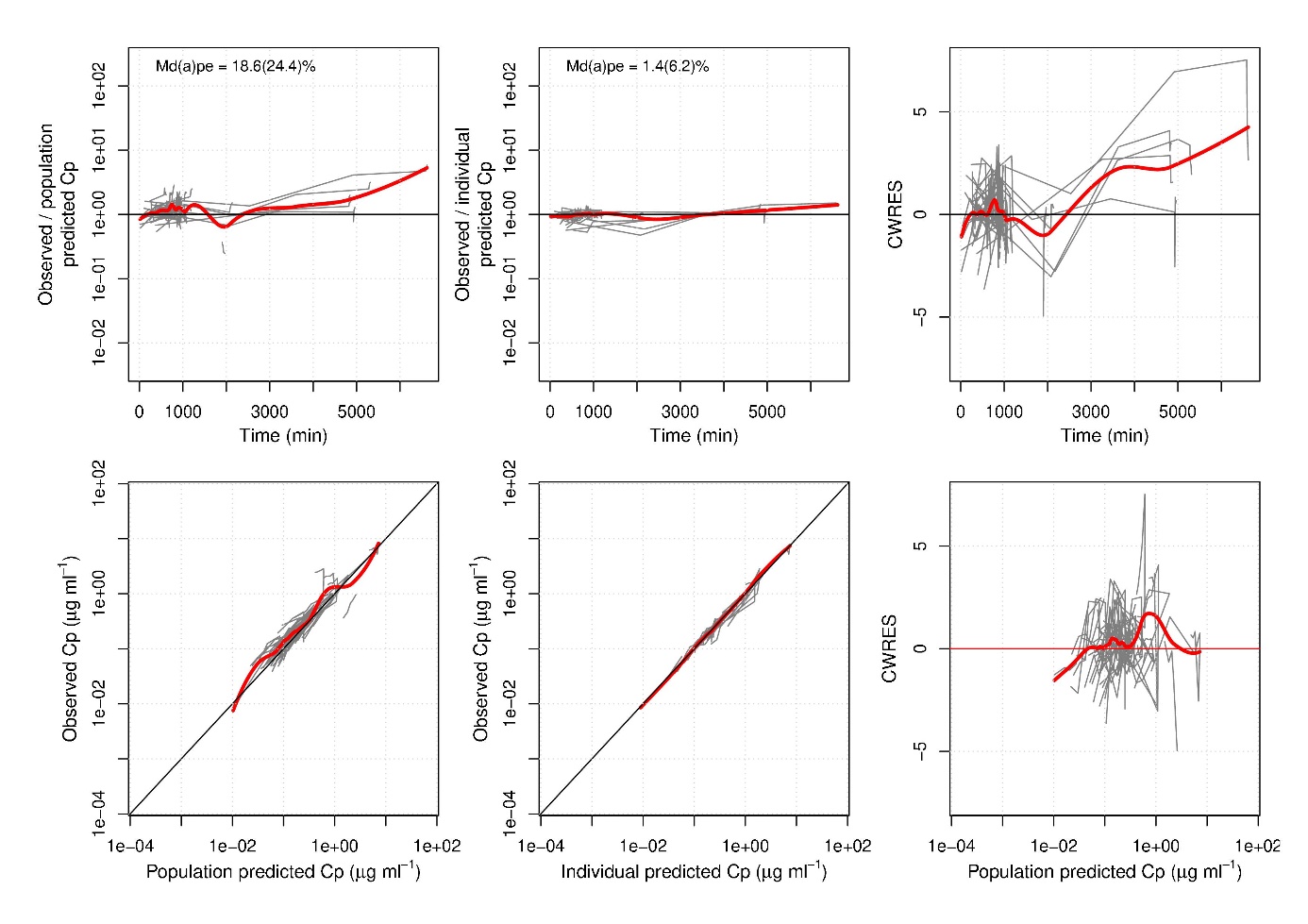


Supplementary Figure 15: Diagnostic plots for the adjusted posthoc PK model to predict remimazolam arterial samples for patients being treated in the ICU. The red line shows a LOESS smoother.


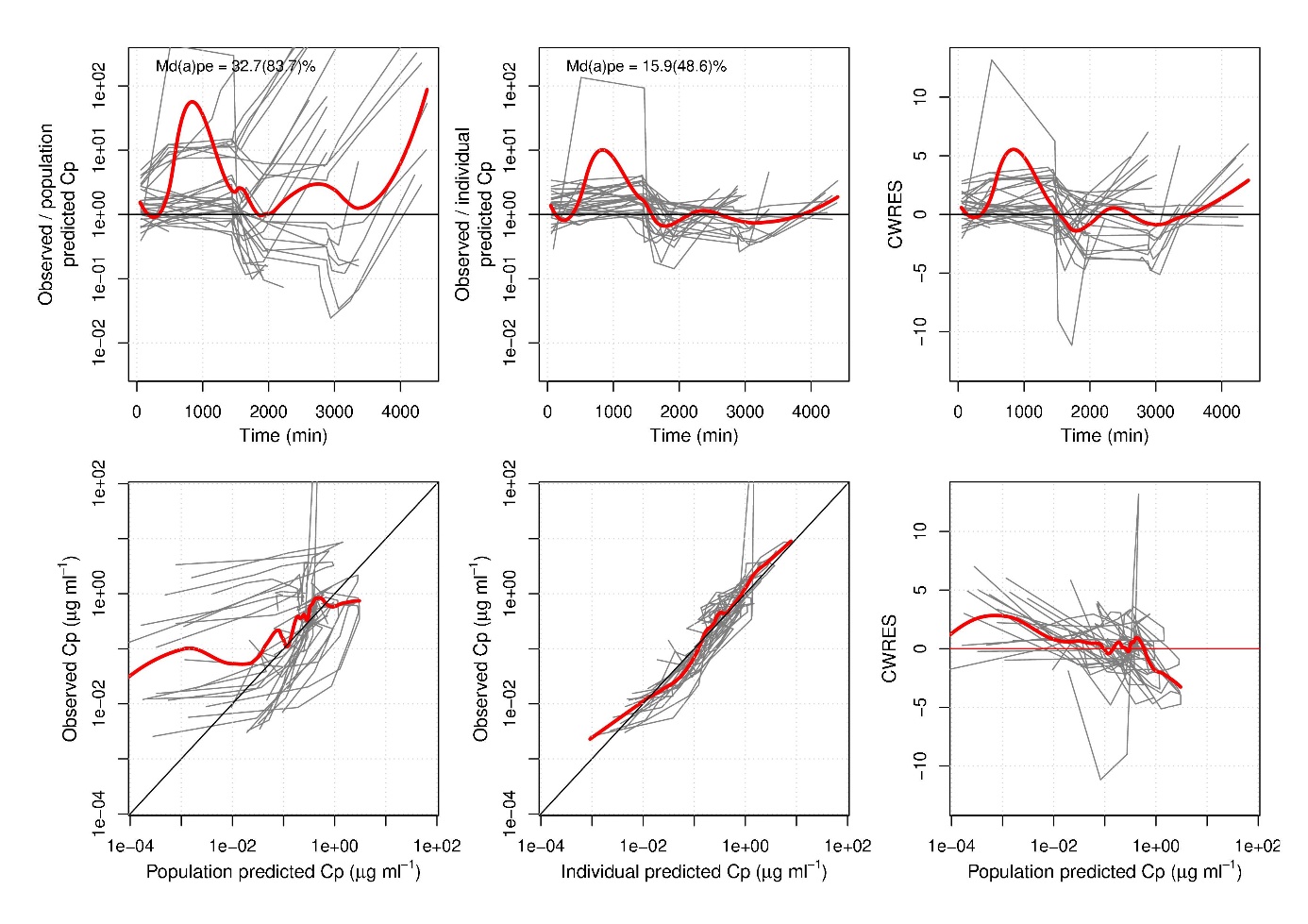


Supplementary Figure 16: Diagnostic plots for the adjusted posthoc PK model to predict remimazolam venous samples for patients being treated in the ICU. The red line shows a LOESS smoother.


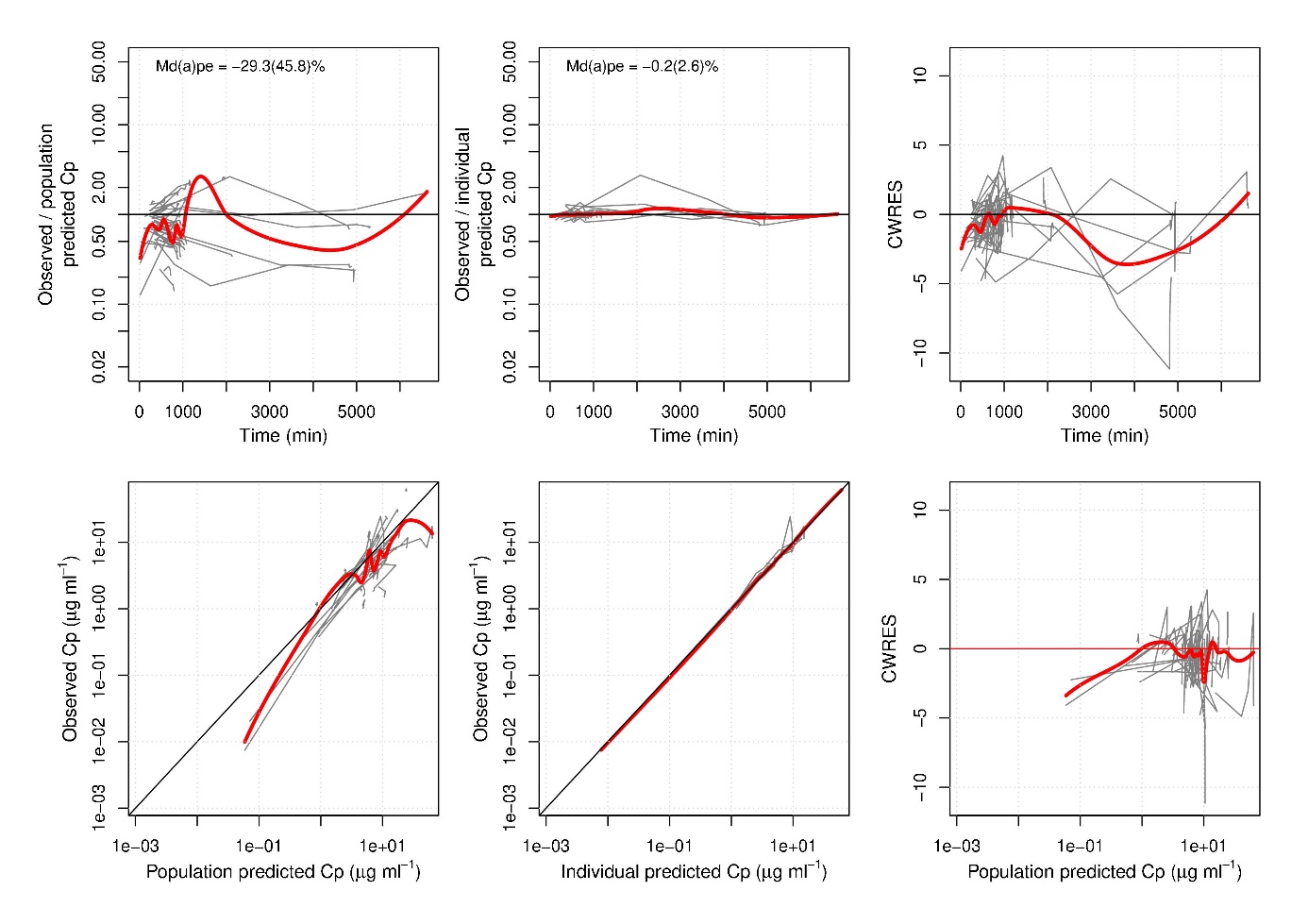


Supplementary Figure 17: Diagnostic plots for the adjusted posthoc PK model to predict CSN7054 arterial samples for patients being treated in the ICU. The red line shows a LOESS smoother.


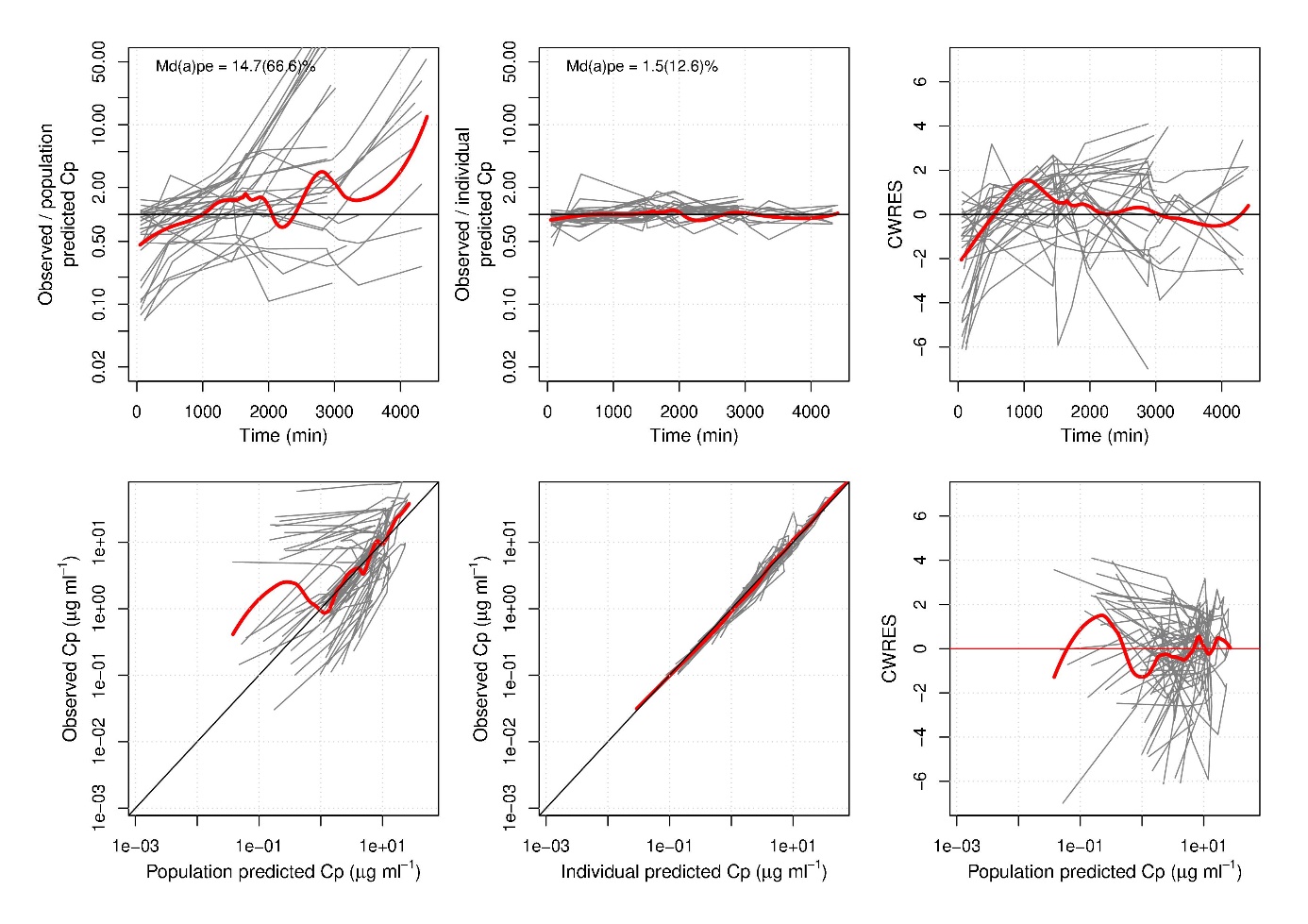


Supplementary Figure 18: Diagnostic plots for the adjusted posthoc PK model to predict CNS7054 venous samples for patients being treated in the ICU. The red line shows a LOESS smoother.


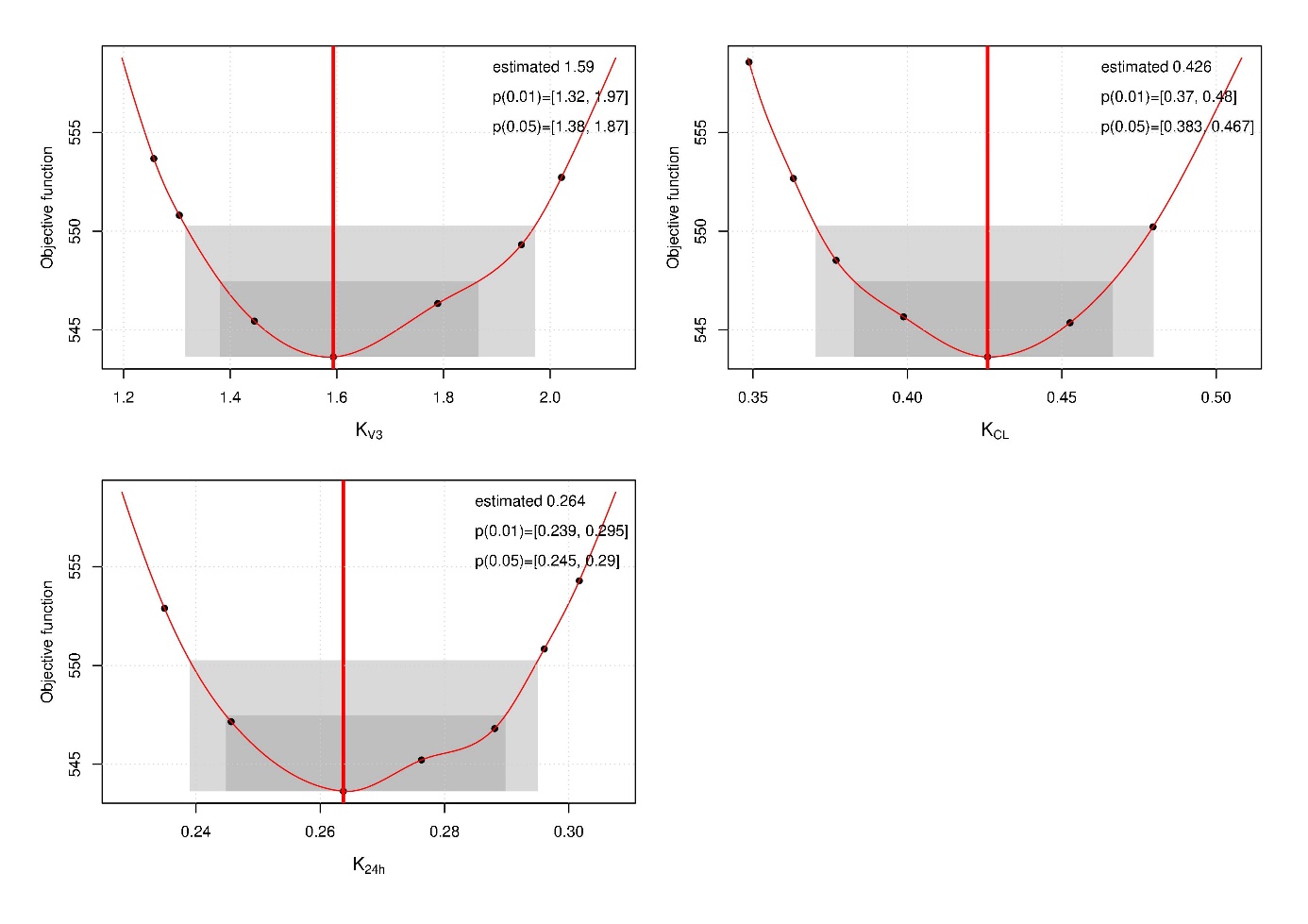


Supplementary Figure 19: Likelihood profiles for the PK model adjustments for patients being treated in the ICU.


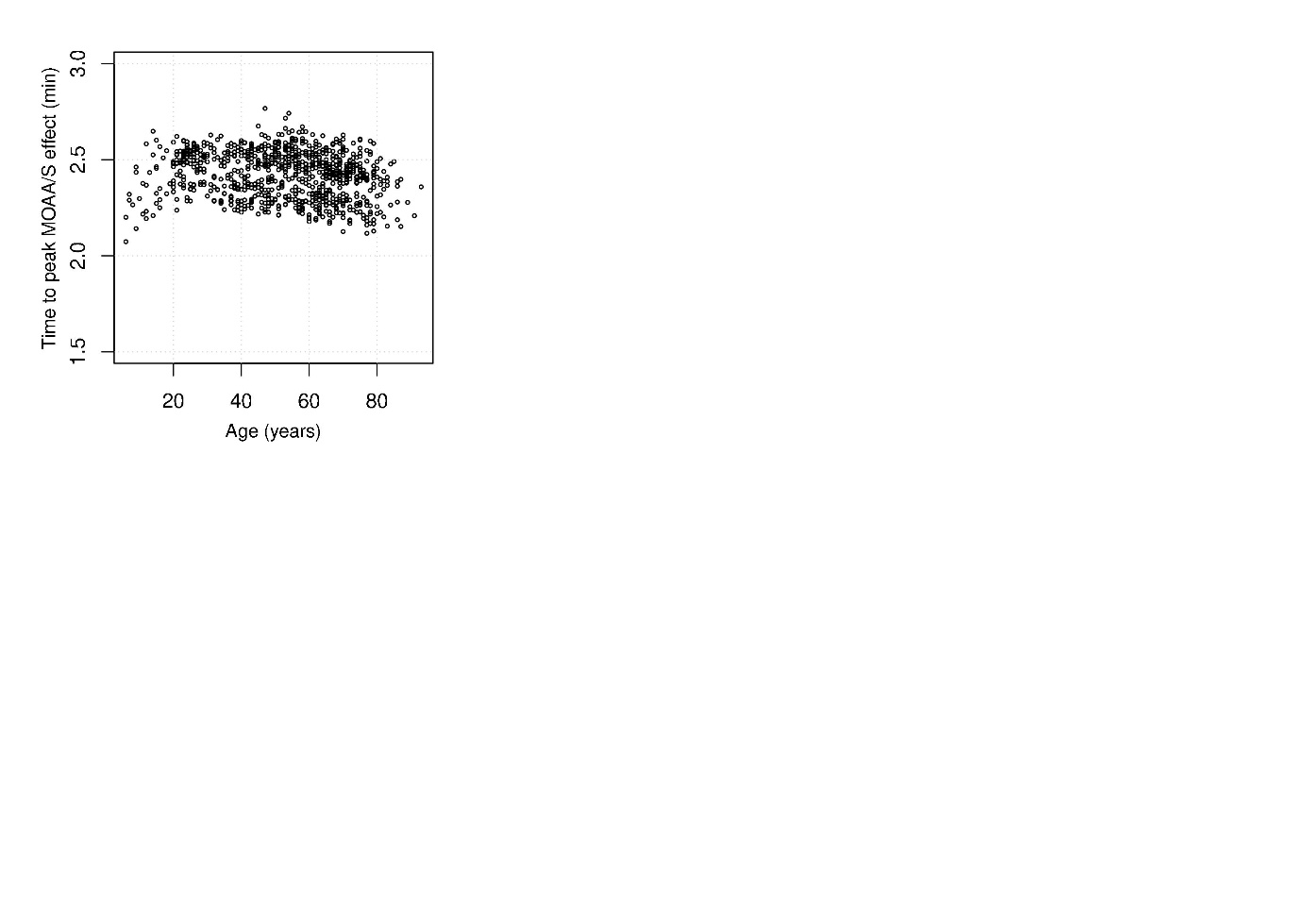


Supplementary Figure 20: Predicted time to peak MOAA/S effect.
